# Synthesis and Some Coordination Chemistry of Phosphane-Difunctionalized Bis(amidinato)-Heavier Tetrylenes: A Previously Unknown Class of PEP Tetrylenes (E = Ge and Sn)

**DOI:** 10.1021/acs.inorgchem.3c01953

**Published:** 2023-09-11

**Authors:** Javier A. Cabeza, Felipe García, Pablo García-Álvarez, Rubén García-Soriano, Enrique Pérez-Carreño

**Affiliations:** † Departamento de Química Orgánica e Inorgánica, Centro de Innovación en Química Avanzada ORFEO−CINQA, Universidad de Oviedo, E-33071 Oviedo, Spain; ‡ School of Chemistry, Monash University, Clayton, Victoria 3800, Australia; § Departamento de Química Física y Analítica, Universidad de Oviedo, E-33071 Oviedo, Spain

## Abstract

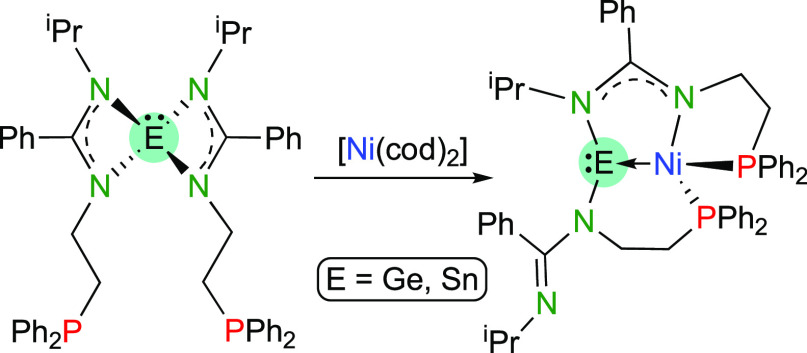

The bis(amidinato)-heavier tetrylenes E(bzamP)_2_ (E = Ge (**2a**) and Sn (**2b**); bzamP = *N*-isopropyl-*N*′-(diphenylphosphanylethyl)benzamidinate), which are equipped with one heavier tetrylene (germylene or stannylene) and two phosphane fragments (one on each amidinate moiety) as coordinable groups, have been synthesized from the benzamidinum salt [H_2_bzamP]Cl and GeCl_2_(dioxane) or SnCl_2_ in 2:1 mol ratio. A preliminary inspection of their coordination chemistry has shown that their amidinate group can also be involved in the bonding with the metal atoms as tridentate ENP and tetradentate PENP′ coordination modes have been observed for the ECl(bzamP)_2_ ligand of [Ir{κ^3^*E*,*N*,*P*-ECl(bzamP)_2_}(cod)] (E = Ge (**3a**) and Sn (**3b**); cod = η^4^-1,5-cyclooctadiene) and the E(bzamP)_2_ ligand of [Ni{κ^4^*E*,*N*,*P*,*P*′-E(bzamP)_2_}] (E = Ge (**4a**) and Sn (**4b**)), which are products of reactions of **2a** and **2b** with [IrCl(cod)]_2_ (1:0.5 mol ratio) and [Ni(cod)_2_] (1:1 mol ratio), respectively. These products contain a 5-membered NCNEM ring that results from the insertion of the metal M atom into an E–N bond of **2a** and **2b**. Additionally, while iridium(I) complexes **3a** and **3b** are chloridotetryl derivatives (insertion of the tetrylene E atom into the Ir–Cl bond has also occurred) that have an uncoordinated phosphane group, nickel(0) complexes **4a** and **4b** contain a tetrylene fragment that, maintaining the lone pair, behaves as a σ-acceptor (*Z*-type) ligand.

## Abstract 2

A new class of heavier tetrylenes featuring two phosphane-functionalized amidinate groups has been prepared and their coordination chemistry has started to be investigated. The insertion of a metal atom into one of their E−N bonds has been observed in iridium(I) and nickel(0) complexes. The latter contain a σ-acceptor (*Z*-type) tetrylene ligand.

## 1 Introduction

**Figure 1:**
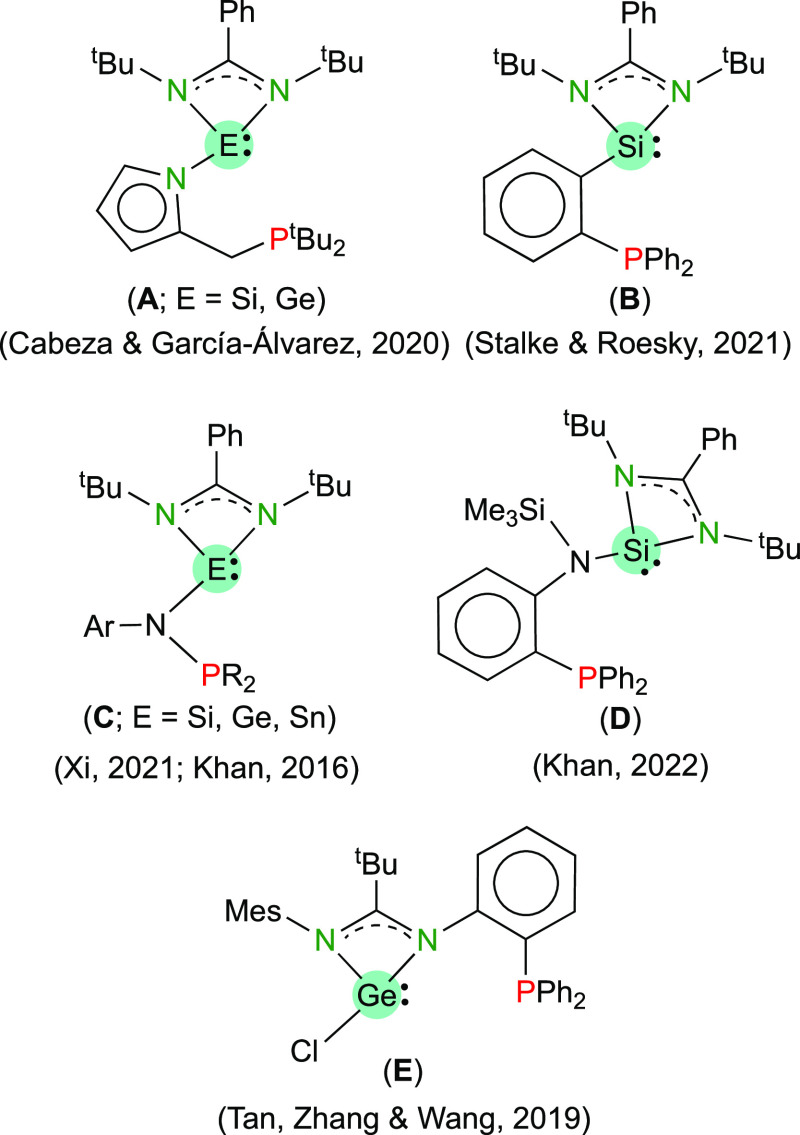
Currently known phosphane-functionalized amidinatotetrylenes.

Heavier tetrylenes (HTs),^1^ which are the heavier analogues of carbenes (silylenes, germylenes, stannylenes, and plumbylenes), have been used as ligands in transition-metal chemistry^2,3^ since the 1970s.^4^ However, their incorporation into bi- or tridentate ligands is much more recent.^5–13^ Within the broad family of bi- and tridentate HT ligands, those containing at least one amidinato-HT (in which the E atom is chelated by the N atoms of the amidinate group^7^) have recently stood out since some of their complexes have shown remarkable catalytic activities.^6–12^ Most of these species have been prepared by replacing the Cl atom of amidinato-HTs of the type ECl(RNC(R′)NR) (E = heavier tetrel atom) with the appropriate functionalized fragment. This is the case for the recently reported HTs **A**,^8^**B**,^9^**C**,^10^ and **D**(11) (Figure 1), which are the only currently known amidinato-HTs comprising a phosphane fragment dangling from the E atom. The attachment of a coordinable fragment to a N atom of the amidinato-HT group is very rare, with compound **E** (Figure 1) being the only phosphane-functionalized amidinato-HT of this type known to date.^12^ Although various HTs decorated with two phosphane groups, rendering PEP pincer-type ligands (E = Ge and Sn), have been previously reported,^13^ the functionalization of an amidinato-HT with two phosphane groups is currently unknown.

**Figure 2:**
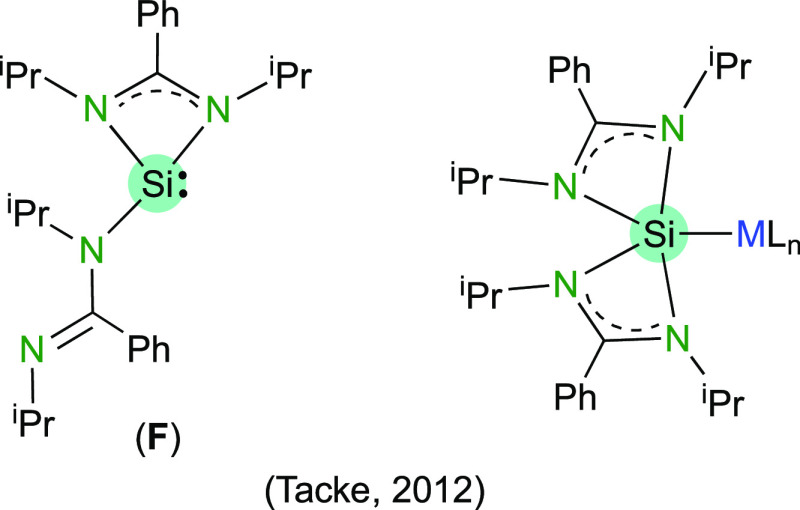
Tacke’s bis(amidinato)tetrylene (**F**) and its coordination to transition-metal complexes.

A particular family of amidinato-HTs is constituted of those that contain two amidinate groups. These compounds, which are described by the general formula E{RNC(R′)NR}_2_ (E = Si,^14^ Ge,^15^ Sn,^16^ and Pb^17^), display one or both amidinate groups chelating the E atom, depending on the E atom and the R and R′ substituents. Notably, upon attachment to a transition metal, the two amidinate groups of bis(amidinato)silylenes generally chelate the Si atom (Figure 2).^14a,18^ The coordination chemistry of bis(amidinato)germylenes and bis(amidinato)stannylenes remains underinvestigated.^19^

This contribution, aiming at expanding the family of polydentate amidinato-HT ligands and their coordination chemistry, describes the synthesis of an amidinium cation equipped with a phosphane functionality on an N atom, namely, the cation of [H_2_bzamP]Cl (**1**; HbzamP = *N*-isopropyl-*N*′-diphenylphosphanyl ethyl)benzamidine), and its use as a precursor to unprecedented bis(amidinato)-HTs of type E(bzamP)_2_, E = Ge (**2a**) and Sn (**2b**), which have a pendant phosphane group on each amidinate fragment. This article also reports the incorporation of these potentially tridentate PEP bis(amidinato)-HT ligands into iridium(I) and nickel(0) complexes.

## 2 Results and Discussion

**Scheme 1:**
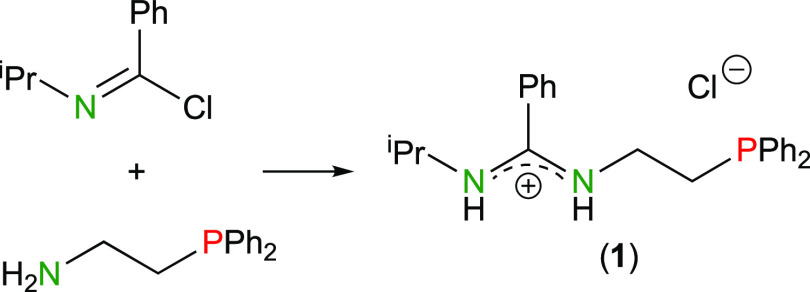
Synthesis of Compound **1**

The phosphane-functionalized benzamidinium salt [H_2_bzamP]Cl (**1**) was prepared by treating *N*-isopropylbenzimidoyl chloride with 2-(diphenylphosphanyl)ethylamine (Scheme 1), following the method previously reported by Zhong and Zhu’s group to synthesize amidine-phosphanes,^20^ but using the appropriate aminophosphane. The asymmetry of the benzamidinium cation of **1** was clearly evidenced by its ^1^H NMR spectrum, which shows two N*H* signals (δ = 10.65 and 10.54 ppm). As far as we are aware, only a handful of related phosphane-functionalized amidines have been previously reported.^20,21^

**Scheme 2:**
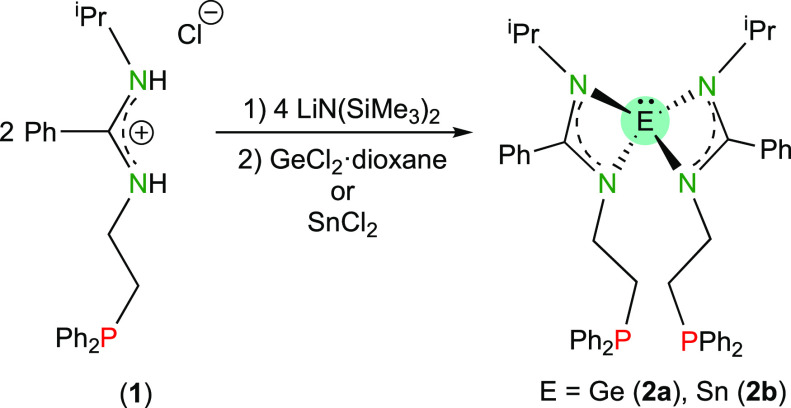
Synthesis of Compounds **2a** and **2b**

Deprotonation of **1** with LiN(SiMe_3_)_2_ followed by the addition of GeCl_2_(dioxane) or SnCl_2_, in 2:4:1 mol proportion, allowed the synthesis of the bis(amidinato)-HTs E(bzamP)_2_ (E = Ge (**2a**) and Sn (**2b**); Scheme 2), which were isolated in yields of 29% (**2a**) and 80% (**2b**). Notably, a mechanochemical approach^22^ (ball milling the solid reagents for 90 min at 1800 rpm) allowed a higher yield of **2a** (56%).

**Figure 3:**
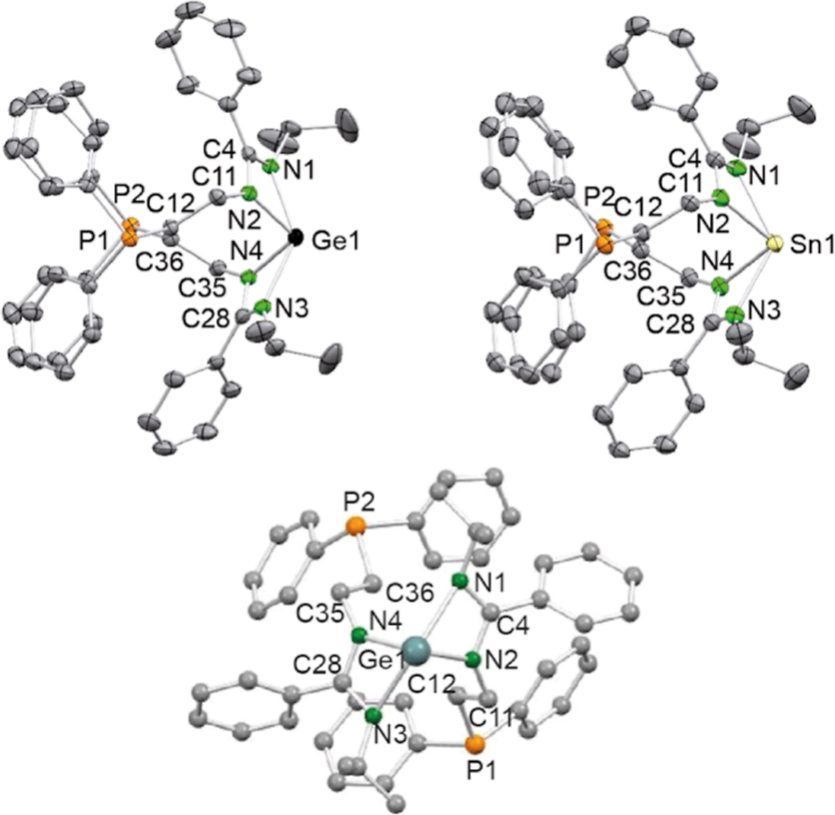
SCXRD molecular structures of **2a** (top left) and **2b** (top right) (30% displacement ellipsoids, H atoms omitted for clarity) and a view of **2a** along the noncrystallographic *C*_2_ axis (bottom; ball and stick representation, H atoms omitted for clarity). Only one of the two independent but analogous molecules found in the asymmetric unit of **2a** is shown. Selected interatomic distances (Å) and angles (deg): **2a**: Ge1–N1 2.239(4), Ge1–N2 1.989(4), Ge1–N3 2.285(4), Ge1–N4 1.974(4), C4–N1 1.318(6), C4–N2 1.337(6), C11–N2 1.450(6), C11–C12 1.527(6), C12–P1 1.843(4), C28–N3 1.320(6), C28–N4 1.337(6), C35–N4 1.451(6), C35–C36 1.527(6), and C36–P2 1.837(4) and N1–Ge1–N2 62.2(2), N1–Ge1–N3 139.5(1), N1–Ge1–N4 92.0(2), N2–Ge1–N3 91.5(2), N2–Ge1–N4 101.1(2), N3–Ge1–N4 61.7(1), N1–C4–N2 111.5(4), and N3–C28–N4 111.8(4). **2b**: Sn1–N1 2.407(3), Sn1–N2 2.184(3), Sn1–N3 2.358(3), Sn1–N4 2.186(2), C4–N1 1.315(4), C4–N2 1.338(4), C11–N2 1.452(4), C11–C12 1.525(4), C12–P1 1.848(3), C28–N3 1.317(4), C28–N4 1.324(4), C35–N4 1.450(4), C35–C36 1.531(4), and C36–P2 1.840(3) and N1–Sn1–N2 57.69(9), N1–Sn1–N3 128.33(9), N1–Sn1–N4 87.23(9), N2–Sn1–N3 87.51(9), N2–Sn1–N4 96.86(9), N3–Sn1–N4 57.97(9), N1–C4–N2 113.8(3), and N3–C28–N4 113.4(3).

Both **2a** and **2b** were characterized by single-crystal X-ray diffraction (SCXRD). Both compounds are isostructural (Figure 3, top). The two benzamidinate groups chelate the corresponding E atom through their N atoms in such a way that the molecules present a noncrystallographic *C*_2_ symmetry with the pseudo-2-fold axis containing the E atom and its corresponding lone pair orbital (Figure 3, bottom). No interaction between the phosphane P atoms and the tetrylene E atom is observed. If one considers that the E lone pair of **2a** and **2b** occupies a coordination position, these compounds have their N atoms in 4 vertexes of a 5-vertex polyhedron. The XRD structures of **2a** and **2b** show (Figure 3) that the coordination geometry of the E atom is distorted square pyramidal, as has been previously observed for other bis(amidinato)tetrylenes of type E{RNC(R′)NR}_2_ (E = Ge^15^ and Sn^16^).

The room-temperature NMR spectra of **2a** and **2b** indicate that their bzamP groups are equivalent in solution, also confirming that both phosphane groups remain uncoordinated (NMR, δ_31P_ = −21.5 (**1**), −21.7 (**2a**), and −21.8 (**2b**) ppm, all singlets). The ^119^Sn chemical shift of **4b** (−154 ppm) is comparable to those of other bis(amidinato)stannylenes.^16^ The fact that the room temperature ^1^H NMR spectrum of **2a** is rather broad (Figure S3-top) led us to acquire its ^1^H NMR and ^31^P{^1^H} spectra at lower temperatures. The room-temperature signal of the isopropyl methyl groups broadens at lower temperatures and is split into two signals at −80 °C (Figure S3a), indicating that the facile rotation of the ^i^Pr groups about the ^i^Pr–N bond at room temperature is restricted at very low temperatures. The signals corresponding to the CH_2_CH_2_ protons also broaden at lower temperatures. The ^31^P{^1^H} NMR spectrum is a singlet in the range of 25 to −80 °C (Figure S4a), indicating that both P atoms are equivalent and pendant at all temperatures.

**Scheme 3:**
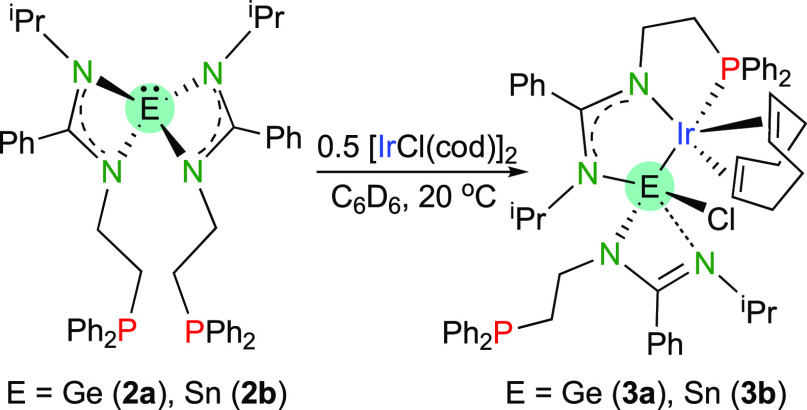
Synthesis of Compounds **3a** and **3b**

Given the novelty of **2a** and **2b** and their potential as tridentate PEP ligands, we decided to investigate their reactivity toward transition-metal complexes. Both tetrylenes reacted quickly at room temperature with [IrCl(cod)]_2_ (1:0.5 mol ratio) to produce [Ir{κ^3^*E*,*N*,*P*-ECl(bzamP)_2_}(cod)] (E = Ge (**3a**) and Sn (**3b**); Scheme 3) almost quantitatively. Both compounds were isolated as yellow solids after washing with *n*-hexane, a solvent that could not be totally removed after drying the solid for several hours at reduced pressure. SCXRD analyses confirmed that both **3a** and **3b** coprecipitate with solvents such as *n*-hexane or diethyl ether (see Supporting Information).

**Figure 4:**
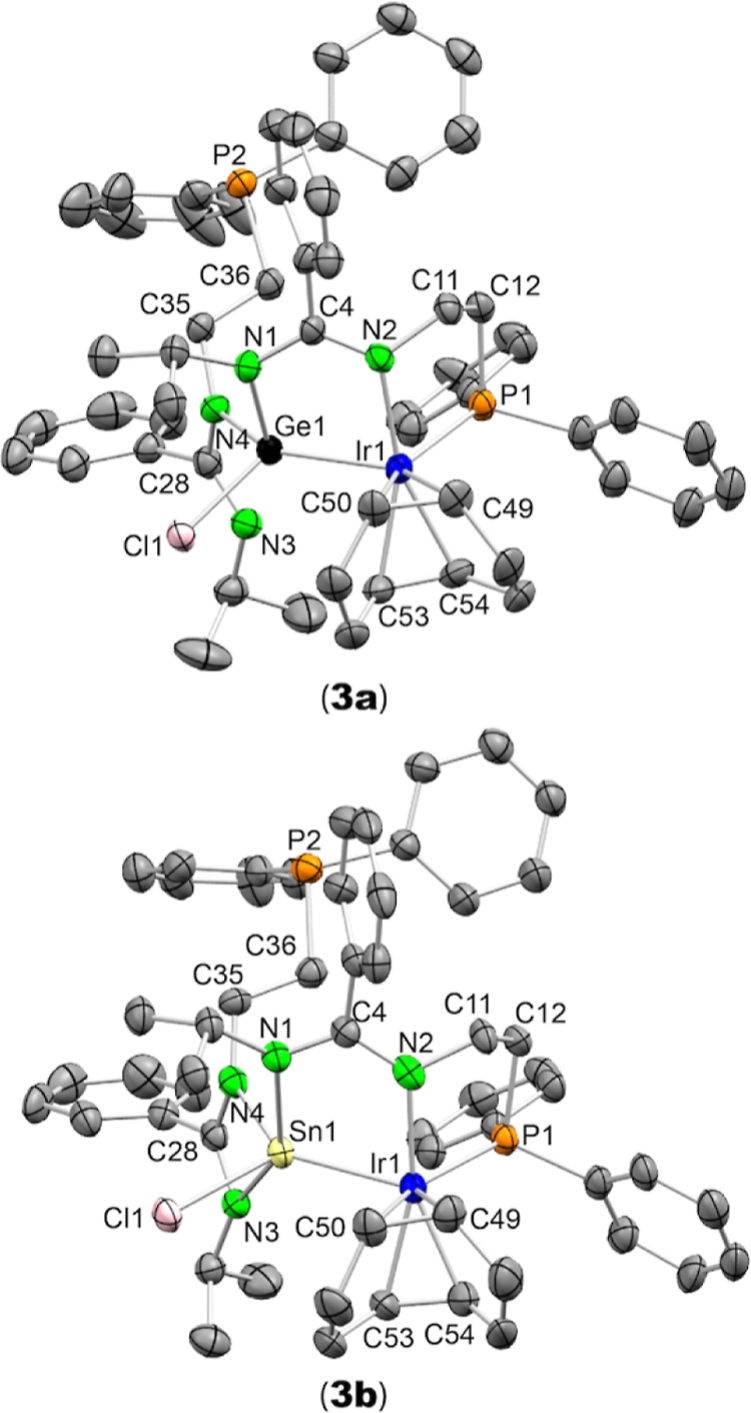
SCXRD molecular structures of **3a** (top) and **3b** (bottom) (30% displacement ellipsoids; H atoms have been omitted for clarity). Selected interatomic distances (Å) and angles (deg): **3a**: Ge1–N1 1.997(6), Ge1–Ir1 2.4027(8), Ge1···N3 2.708(6), Ge1–N4 1.906(6), Ge1–Cl1 2.247(2), Ir1–N2 2.095(6), Ir1–P1 2.297(2), Ir1–C49 2.193(6), Ir1–C50 2.147(7), Ir1–C53 2.180(8), Ir1–C54 2.190(7), C4–N1 1.359(9), C4–N2 1.301(9), C11–N2 1.482(9), C11–C12 1.53(1), C12–P1 1.836(8), C28–N3 1.27(1), C28–N4 1.38(1), C35–N4 1.460(9), C35–C36 1.52(1), and C36–P2 1.853(8) and N1–Ge1–N4 97.7(3), N1–Ge1–Ir1 95.0(2), N4–Ge1–Ir1 139.2(2), N4–Ge1–Cl1 96.9(2), N1–Ge1–Cl1 100.3(2), Cl1–Ge1–Ir1 118.74(5), Ge1–Ir1–P1 110.84(5), Ge1–Ir1–N2 79.9(2), P1–Ir1–N2 82.0(2), N1–C4–N2 121.8(6), and N3–C28–N4 116.9(7). **3b**: Sn1–N1 2.246(7), Sn1–Ir1 2.5715(7), Sn1–N3 2.465(7), Sn1–N4 2.110(8), Sn1–Cl1 2.422(2), Ir1–N2 2.126(8), Ir1–P1 2.284(2), Ir1–C49 2.16(1), Ir1–C50 2.18(1), Ir1–C53 2.148(9), Ir1–C54 2.15(1), C4–N1 1.33(1), C4–N2 1.35(1), C11–N2 1.47(1), C11–C12 1.54(1), C12–P1 1.854(9), C28–N3 1.29(1), C28–N4 1.36(1), C35–N4 1.43(1), C35–C36 1.52(1), and C36–P2 1.855(9) and N1–Sn1–N4 91.3(3), N1–Sn1–Ir1 88.4(2), N4–Sn1–Ir1 138.9(2), N4–Sn1–Cl1 100.0(2), N1–Sn1–Cl1 94.9(2), Cl1–Sn1–Ir1 121.03(7), N1–Sn1–N3 147.7(3), N4–Sn1–N3 57.9(3), N3–Sn1–Ir1 120.8(2), Cl1–Sn1–N3 82.2(2), Sn1–Ir1–P1 109.23(6), Sn1–Ir1–N2 81.2(2), P1–Ir1–N2 82.2(2), N1–C4–N2 121.2(7), and N3–C28–N4 115.4(8).

The molecular structures of **3a** and **3b** are very similar (Figure 4). They contain a 5-membered NCNEIr ring that results from the insertion of the Ir atom into an E–N bond of **2a** and **2b**. The phosphane fragment of the bzamP group involved in this insertion is also coordinated with the Ir atom. An Ir to E atom transfer of the Cl atom has also occurred. Accordingly, the original tetrylene fragment has been transformed into a chloridotetryl ligand. The ligand environment of the Ir atoms is better described as distorted trigonal bipyramidal (calculated τ_5_ values^23^ are 0.76 for **3a** and 0.63 for **3b**), in which the coordinated N atom (N2) and one of the cod olefinic fragments (C53 and C54) are in a pseudoaxial position. The Ge–Ir bond distance of **3a**, 2.4027(8) Å, compares well with those found in complexes containing PGeP germyl Ge–Ir bonds (2.4275(3) Å for a cod complex and 2.3888(5) Å for a dicarbonyl derivative).^5k^ Although a PSnP stannyl Ir complex has been previously described,^5l^ its SCXRD structure has not been reported. The most notable structural difference between **3a** and **3b** is the attachment of the amidinate N atoms of the other bzamP group to the corresponding E atom, which is more asymmetric in **3a** (Ge1–N3 2.708(6) Å, Ge1–N4 1.906(6) Å) than in **3b** (Sn1–N3 2.465(7) Å, Sn1–N4 2.110(8) Å), reflecting the congested environment of the E atoms and the smaller size of germanium (vs tin). In both **3a** and **3b**, the Ir atom is pentacoordinate. While the insertion of a tetrylene E atom into a Cl–M bond (to become a tetryl ligand) has been often observed,^7,13,24^ the insertion of a metal atom M into a N–E bond of an amidinato- or guanidinato-HT is less frequent.^25–29^ As far as we are aware, ENP (E = Ge and Sn) pincer ligands, such as those of **3a** and **3b**, have no precedent in the chemical literature.

**Scheme 4:**
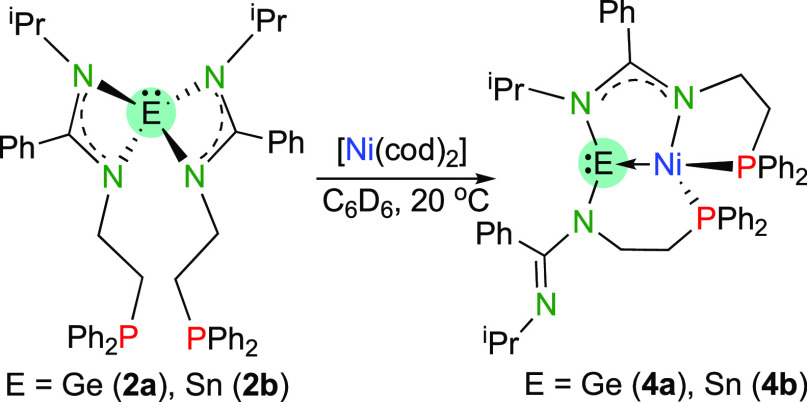
Synthesis of Compounds **4a** and **4b**

With the aim of preventing the conversion of the tetrylene fragments into tetryl ligands upon the reactions of **2a** and **2b** with transition-metal complexes, [Ni(cod)_2_] was chosen as a chlorido-free metal precursor. Both **2a** and **2b** reacted quickly with [Ni(cod)_2_] (1:1 mol ratio) at room temperature. NMR monitoring of reactions performed in C_6_D_6_ showed the almost quantitative formation of compounds [Ni{κ^4^*E*,*N*,*P*,*P*′-E(bzamP)_2_}] (E = Ge (**4a**) and Sn (**4b**); Scheme 4) after 30 min. However, compound **4b** always decomposed during the reaction workup and it could not be isolated as a pure solid.

The ^1^H, ^13^C{^1^H}, and ^31^P{^1^H} NMR spectra of both compounds are very similar, indicating that they are isostructural and asymmetric (they have two different bzamP groups), that they do not contain cod, and that both P atoms of their E(bzamP)_2_ ligand are coordinated (δ_31P_ for **4a** = 43.7 and 26.7 ppm (*J*_P–P_ = 27 Hz) and δ_31P_ for **4b** = 37.1 and 18.3 ppm (*J*_P–P_ = 32 Hz)). The ^119^Sn{^1^H} NMR spectrum of **4b** is a broad signal at 455 ppm. As expected, the ^119^Sn chemical shift of **4b**, which contains a coordinated disubstituted *Z*-type stannylene, differs considerably from those of **3b** (−262 ppm), which contains a coordinated tetrasubstituted stannyl ligand, and **2b** (−154 ppm), which is an uncoordinated bis(amidinato)stannylene. The ^119^Sn chemical shifts of free and coordinated stannylenes and stannyl ligands strongly depend upon the number and basicity of the groups attached to the tin atom (the greater the number and the basicity of these groups, the greater the shielding) and also on the nature of the metal atom (when coordinated).^30^ Coordinated disubstituted *Z*-type stannylenes appear at positive ^119^Sn chemical shifts (in the range 671–155 ppm for Ni^0^ and Pd^0^ complexes).^5o^

**Figure 5:**
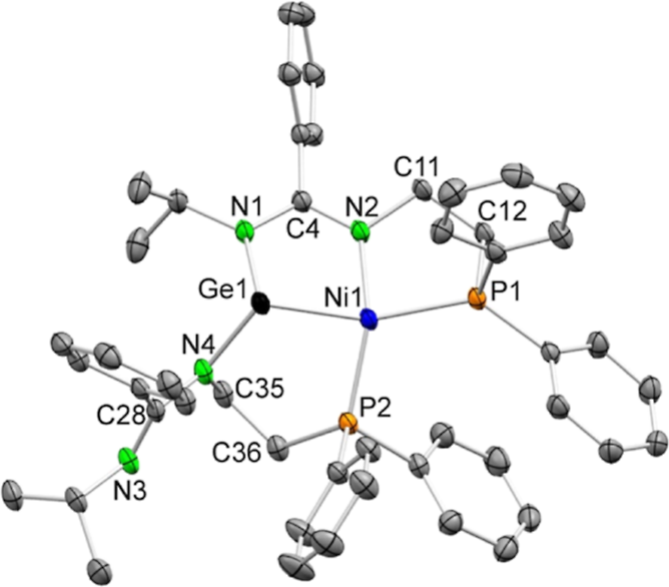
SCXRD molecular structure of **4a** (30% displacement ellipsoids; H atoms have been omitted for clarity). Selected interatomic distances (Å) and angles (deg): Ge1–N1 2.010(2), Ge1–Ni1 2.2910(4), Ge1–N4 1.934(2), Ni1–N2 1.922(2), Ni1–P1 2.2163(6), Ni1–P2 2.1189(7), C4–N1 1.335(3), C4–N2 1.333(3), C11–N2 1.492(3), C11–C12 1.522(4), C12–P1 1.839(2), C28–N3 1.284(3), C28–N4 1.383(3), C35–N4 1.459(3), C35–C36 1.522(4), and C36–P2 1.855(2) and N1–Ge1–N4 97.37(8), N1–Ge1–Ni1 91.62(6), N4–Ge1–Ni1 109.04(6), N2–Ni1–P2 145.99(7), N2–Ni1–P1 88.53(6), P2–Ni1–P1–112.47(3), N2–Ni1–Ge1 85.49(6), P2–Ni1–Ge1–89.31(2), P1–Ni1–Ge1 147.22(3), N1–C4–N2 119.4(2), and N3–C28–N4 120.3(2).

Compound **4a** was characterized by SCXRD. Figure 5 shows that the Ni atom has been inserted into a Ge–N bond of **2a** and is also attached to the phosphane fragment of the bzamP group involved in this insertion. The Ni atom is also attached to the P atom of the other bzamP group, which is bonded to the Ge atom only through the amidinate N atom that bears the phosphane fragment. The Ge–Ni bond distance, 2.2910(4) Å, is slightly shorter than those reported for other *Z*-type bonded germylene–nickel(0) complexes (2.217(1),^5s^ 2.2786(3),^5t^ 2.285(1),^5u^ and 2.2559(4) Å^12^). Overall, the coordination environment of the Ge atom is pyramidal, whereas that of the Ni atom (τ_4_ = 0.47) is 47% along the way that goes from square planar (τ_4_ = 0) to tetrahedral (τ_4_ = 1).^31^ As far as we are aware, only one tetradentate PGeNP′ ligand, such as that of **4a**, has been previously reported: a related nickel(0) complex was serendipitously prepared in low yield by reacting two equivalents of the monophosphane-functionalized chlorido(amidinato)germylene **E** (Figure 1) with [Ni(cod)_2_] and reducing the resulting [Ni(κ^2^*Ge*,*P*-**E**)_2_] product with potassium metal.^12^ No PSnNP ligand, such as **4b**, had been reported prior to this work.

**Figure 6:**
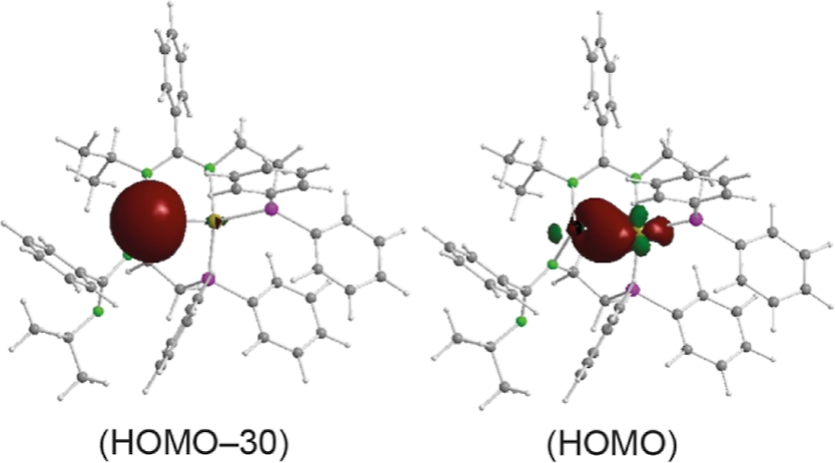
Selected NBOs of **4a** (structure optimized at the wB97XD/SDD_Ni,Ge_/cc-pVDZ level of theory), showing the Ge lone pair orbital (HOMO – 30) and the orbital responsible for the Ge ← Ni *Z*-type interaction (HOMO).

**Figure 7:**
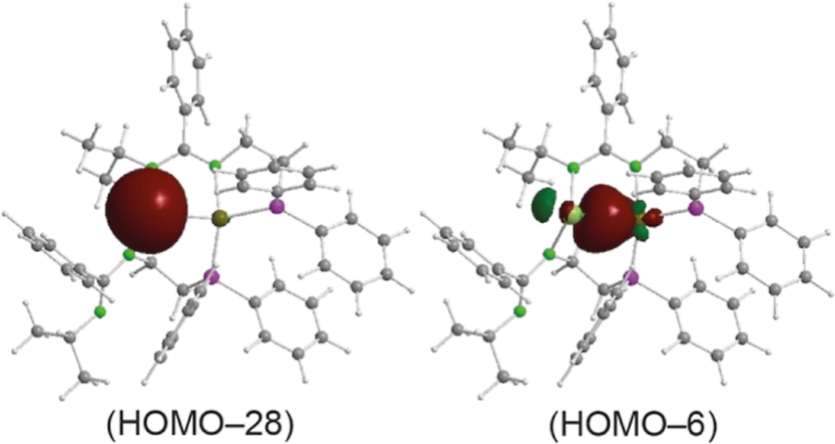
Selected NBOs of **4b** (structure optimized at the wB97XD/SDD_Ni,Sn_/cc-pVDZ level of theory), showing the Sn lone pair orbital (HOMO – 28) and the orbital responsible for the Sn ← Ni *Z*-type interaction (HOMO – 6).

The coordination environment of the Ge atom of complex **4a** suggested the presence of a lone pair on the Ge atom and, therefore, that the Ni atom should donate σ-electron density to the Ge atom. This hypothesis was corroborated by DFT-NBO calculations (at the wB97XD/SDD_Ni,Ge_/cc-pVDZ level of theory). Figure 6 clearly shows that the germylene maintains its lone pair (HOMO – 30; 76.2% s, 23.8% p) and that the HOMO is a σ-type orbital that results from the overlap of a filled spd hybrid (29.6% s, 12.8% p, 57.6% d) of the Ni atom with an empty sp hybrid (11.3% s, 88.2% p, 0.5% d) of the Ge atom. A similar calculation with the tin–nickel complex **4b** (at the wB97XD/SDD_Ni,Sn_/cc-pVDZ level of theory) (Figure 7) indicated that the tin lone pair is the HOMO – 28 orbital (85.6% s, 14.4% p) and that the orbital responsible for the σ-type interaction (HOMO – 6) results from the overlap of a filled spd hybrid (25.2% s, 45.7% p, 29.1% d) of the Ni atom with an empty spd hybrid (5.3% s, 94.4% p, 2.3% d) of the Sn atom. Therefore, the tetrylene moieties of complexes **4a** and **4b** behave as σ-acceptor (*Z*-type) ligands.^32^ Although still uncommon in coordination chemistry, a few *Z*-ligands involving HTs have already been identified.^5c,5o,5t,5u,12,33^

## 3 Conclusions

In this work, we have expanded the family of polydentate ligands containing germylenes and stannylenes by designing novel phosphane-difunctionalized bis(amidinato)-HTs (**2a** and **2b**). The incorporation of the potentially PEP tridentate proligands **2a** and **2b** to Ir(I) and nickel(0) complexes has been achieved. The structures of complexes [Ir{κ^3^*E*,*N*,*P*-ECl(bzamP)_2_}(cod)] (E = Ge (**3a**) and Sn (**3b**)) and [Ni{κ^4^*E*,*N*,*P*,*P*′-E(bzamP)_2_}] (E = Ge (**4a**) and Sn (**4b**)) have shown that the amidinate N atoms of **2a** and **2b** can also be involved in the bonding with the metal atoms as the ECl(bzamP)_2_ ligand of the Ir(I) complexes **3a** and **3b** is ENP tridentate, whereas the E(bzamP)_2_ ligand of the nickel(0) **4a** and **4b** complexes is PENP′ tetradentate. While ENP (E = Ge and Sn) tridentate ligands have no precedent in the chemical literature, PENP′ tetradentate ligands are very uncommon (only one previous example^12^). It is expected that the coordination chemistry of the PEP-HTs **2a** and **2b** will be extended to many other transition metals, which will present new structural motifs and new physical and chemical properties.

## Supporting Information Available

General procedures, experimental details, characterization data, and NMR spectra (PDF)
Atomic coordinates of the DFT-optimized structures of complexes **4a** and **4b** (XYZ)

## Supplementary Material

ic3c01953_si_001.pdf

ic3c01953_si_002.xyz
