# Supplementary material for: Synthesis and Some Coordination Chemistry of Phosphane-Difunctionalized Bis(amidinato)-Heavier Tetrylenes: A Previously Unknown Class of PEP Tetrylenes (E = Ge and Sn)
Source: Inorg Chem. 2023 Sep 11;62(38):15502–9. doi: 10.1021/acs.inorgchem.3c01953 (PMC10523440; doi:10.1021/acs.inorgchem.3c01953)
Supplement: Supplementary file 1 — ic3c01953_si_001.pdf [file ic3c01953_si_001.pdf]

SUPPORTING INFORMATION

Synthesis and Some Coordination Chemistry of Phosphane-Difunctionalized Bis(Amidinato)-Heavier Tetrylenes: A Previously Unknown Class of PEP Tetrylenes (E = Ge, Sn)

Javier A. Cabeza,[*,†] *Felipe García,*[†,#] *Pablo García-Álvarez,*[*,†] *Rubén García-Soriano*[†] *and Enrique Pérez-Carreño*[‡]

†Departamento de Química Orgánica e Inorgánica, Centro de Innovación en Química Avanzada ORFEO-CINQA, Universidad de Oviedo, E-33071 Oviedo, Spain

#School of Chemistry, Monash University, Clayton, Victoria 3800, Australia

‡Departamento de Química Física y Analítica, Universidad de Oviedo, E-33071 Oviedo, Spain

Materials

Solvents were dried over appropriate desiccating reagents and were distilled under argon before use. Compounds 2-(diphenylphosphanyl)ethylamine[S1] and *N*-*iso*propylbenzimidoyl chloride[S2] were prepared following published procedures and were stored under argon in the drybox. All remaining reagents were purchased from commercial sources. All reagents were stored under argon in a drybox. All reaction products were vacuum-dried for several hours prior to being weighted and analyzed.

Instrumentation and Measurements

All reactions and product manipulations were carried out under argon in an MBraun UNIlab Pro drybox or using Schlenk-vacuum line techniques. Unless otherwise stated, the reactions were carried out at room temperature. Mechanochemical reactions were performed with a Retsch MM400 ball mill using stainless steel (440B type) grinding jars with stainless steel ball bearings, carrying out all reagent and product manipulations in the drybox and sealing the grinding jars with Teflon tape prior to their transfer to the ball mill. NMR spectra were run on Bruker NAV-400, AV-400, and DPX-300 instruments, using as standards the residual protic solvent resonance for ^{1}H [δ(C*H*Cl$_3$) 7.26 ppm; δ(C$_6$*H*D$_5$) 7.16 ppm], the solvent resonance for ^{13}C [δ(*C*$_6$D$_6$) 128.1 ppm; δ(*C*DCl$_3$) 77.2 ppm], external 85% H_3PO_4 in D_2O for ^{31}P (δ 0.0 ppm), and external $SnMe_4$ in $CDCl_3$ for ^{119}Sn (δ 0.0 ppm). Microanalyses were obtained with a Thermo-Finnigan FlashEA112 microanalyzer. High-resolution mass spectra (HRMS) were obtained with a Bruker Impact II mass spectrometer operating in the ESI-Q-TOF positive mode; data given refer to the most abundant isotopomer of the observed species with the greatest mass. CHN microanalyses were not obtained for **1**, **3a** and **3b** since, according to their NMR data, they were affected by minor unknown impurities (for **1**) or variable amounts of *n*-hexane (for **3a** and **3b**), that could not be removed.

Experimental Details and Characterization Data

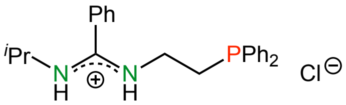

[H$_2$bzamP]Cl (1): A solution of N-*iso*propylbenzimidoyl chloride (1.33 g, 7.30 mmol) in dichloromethane (4 mL) was cooled at –20 °C and dropwise added to a dichloromethane solution (4 mL) of 2-(diphenylphosphanyl)ethylamine (1.67 g, 7.30 mmol) previously cooled at –20 °C. The resulting pale-yellow solution was stirred for 1 h at room temperature. The solvent was then removed *in vacuo* and the solid residue was washed

with hexane (30 mL) to give **1** (almost pure, see Figure S1) as a white solid (2.74 g, 99%). (+)-ESI-Q-TOF: *m/z* 407.1715; calcd. for $C_{25}H_{32}N_2OP$: 407.22521 [*M* – Cl + MeOH]$^+$. ^{1}H NMR ($CDCl_3$, 300.1 MHz, 298 K; Figure S1): δ 10.65 (br s, 1 H, N*H*), 10.54 (d, *J* = 6.0 Hz, 1 H, N*H*), 7.64–7.48 (m, 4 H, C*H*s of Phs), 7.35–7.14 (m, 11 H, C*H*s of Phs), 3.24 (sp, *J* = 6.0 Hz, 1 H, C*H*Me_2), 3.06 (m, 2 H, CH_2C*H_2*P), 2.31 (m, 2 H, C*H_2*CH_2P), 1.19 (d, *J* = 6.0 Hz, 6 H, CH*Me_2*) ppm. ^{13}C{^{1}H} ($CDCl_3$, 75.5 MHz, 298 K; Figure S1): δ 166.0 (s, N*C*N), 136.6 (d, J_{C-P} = 11.6 Hz, *C*s of Phs), 132.7 (s, *C*H of Phs), 132.5 (s, *C*H of Phs), 132.1 (s, *C*H of Ph), 130.0 (s, *C*H of Phs), 129.1 (s, *C*H of Phs), 128.8 (s, *C*H of Ph), 128.7 (s, *C*H of Ph), 126.7 (s, *C*H of Ph), 125.8 (s, *C* of Ph), 47.9 (s, *C*HMe_2), 42.2 (d, J_{C-P} = 27.1 Hz, CH_2*C*H_2P), 29.2 (d, J_{C-P} = 14.9 Hz, *C*H_2CH_2P), 23.3 (s, CH*Me_2*) ppm. ^{31}P{^{1}H} NMR ($CDCl_3$, 121.5 MHz, 298 K; Figure S2): δ –21.9 (s) ppm.

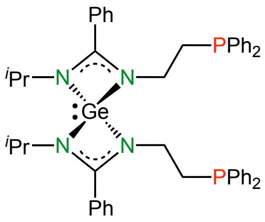

Ge(bzamP)$_2$ (2a): *Method (a):* LiN($SiMe_3$)$_2$ (1.03 g, 6.0 mmol) was added to a toluene/diethylether solution (4:1, 5 mL) of compound **1** (1.23 g, 3.0 mmol) to give an orange suspension that was stirred for 3 h. $GeCl_2$(dioxane) (0.35 g, 1.5 mmol) was added and the resulting suspension was stirred overnight. Hexane (8 mL) was then added, the suspension was filtered through a glass fiber filter and the pale-yellow filtrate was cooled to –20 °C, giving **1** as white crystals (104 mg, 9%). As a considerable amount of solid product accompanied the LiCl, a second batch of **2a** was obtained by washing the filter with hexane/toluene (1:1, 2×10 mL), vacuum-evaporating the pale-yellow filtrate and washing with hexane (4×10 mL) the solid residue (200 mg, 16%). This method provided lower yield and lower product purity than the following mechanochemical method. *Method (b):* A 25 mL mechanochemistry jar was charged with one ball bearing (9/16 in, *i.e.*, 15 mm, 13.62 g), compound **1** (727 mg, 1.8 mmol), LiN($SiMe_3$)$_2$ (592 mg, 3.6 mmol) and $GeCl_2$(dioxane) (205 mg, 0.9 mmol). The solid mixture and was ball-milled for 90 min at 30 Hz. The crude outcome was extracted with toluene (10 mL) and then hexane (10 mL) was added to give a white suspension that was filtered through Celite and the filter was washed with hexane/toluene (1:1, 2×20 mL). The filtrates were collected to give a yellow solution that was evaporated to dryness. The resulting white residue was washed with hexane (5×2 mL) and then with hexane/toluene (10:1, 5×2 mL) to give **1** as a white solid (407 mg, 56%). Anal. (%) calcd. for $C_{48}H_{52}GeN_4P_2$ (*M* = 819.51): C, 70.35; H, 6.40; N, 6.84; found: C, 69.04; H, 6.17; N, 6.66 (possibly affected by the air-sensitivity of the compound, this analysis provides the

best values obtained to date). (+)-ESI-Q-TOF: *m/z* 853.2816; calcd. for $C_{49}H_{57}GeN_4OP_2$: 853.3232 [*M* + H + MeOH]$^+$. ^{1}H NMR (C_6D_6, 300.1 MHz, 298 K; Figure S3): δ 6.78–6.27 (m, 30 H, C*H*s of Phs), 3.80–3.37 (m, 6 H, 2 CH_2CH_2P + 2 C*H*Me_2), 2.46 (br s, 4 H, 2 CH_2CH_2P), 1.30 (br s, 12 H, 2 CH*Me*$_2$) ppm. $^{13}C\{^1H\}$ NMR (C_6D_6, 100.6 MHz, 298 K; Figure S3): δ 167.9 (s, N*C*N), 140.0 (s, *C* of Ph), 133.0 (d, $J_{C\text{-}P}$ = 18.6 Hz, *C*H of Phs), 128.8 (s, *C*H of Ph), 128.7 (d, $J_{C\text{-}P}$ = 8.4 Hz, *C*H of Ph), 127.4–128.5 (m, *C*Hs of Phs), 48.7 (s, *C*HMe$_2$), 44.0 (d, $J_{C\text{-}P}$ = 23.5 Hz, CH_2CH_2P), 33.6 (br s, CH_2CH_2P), 26.2 (s, CH*Me*$_2$) ppm. $^{31}P\{^1H\}$ NMR (C_6D_6, 121.5 MHz, 298 K; Figure S4): δ –21.7 (s) ppm.

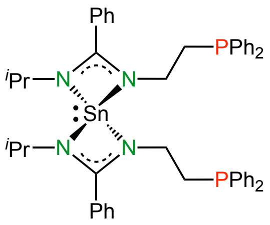

Sn(bzamP)$_2$ (2b): A mixture of compound **1** (411 mg, 1.00 mmol) and $LiN(SiMe_3)_2$ (335 mg, 2.0 mmol) was dissolved in toluene/diethylether (3:1, 4 mL) to give a bright orange suspension that was further stirred for 2.5 h at room temperature. Then, $SnCl_2$ (95 mg, 0.5 mmol) was added. The resulting suspension was stirred overnight at room temperature. All volatiles were removed *in vacuo*, the crude residue was extracted with hexane/toluene (1:8, 4×2 mL) and filtered with the help of a glass-fiber filter and the filtrate was evaporated to dryness. The resulting residue was washed with hexane (3×2 mL) to give **2b** as an off-white solid (345 mg, 80%). Anal. (%) calcd. for $C_{48}H_{52}N_4P_2Sn$ (*M* = 865.61): C, 66.60; H, 6.05; N, 6.47; found: C, 66.99; H, 6.18; N, 5.88. ^{1}H NMR (C_6D_6, 300.1 MHz, 298 K; Figure S5): δ 7.32–7.21 (m, 8 H, C*H*s of Phs), 7.13–6.93 (m, 22 H, C*H*s of Phs), 3.64 (m, 4 H, 2 CH_2CH_2P), 3.54 (sp, *J* = 8.0 Hz, 2 H, 2 C*H*Me$_2$), 2.45 (m, 4 H, 2 CH_2CH_2P), 1.23 (d, *J* = 8.0 Hz, 12 H, 2 CH*Me*$_2$) ppm. $^{13}C\{^1H\}$ NMR (C_6D_6, 75.5 MHz, 298 K; Figure S5): δ 170.7 (s, N*C*N), 140.0 (d, $J_{C\text{-}P}$ = 14.3 Hz, *C* of Ph), 134.3 (s, *C* of Ph), 133.0 (d, $J_{C\text{-}P}$ = 18.9 Hz, *C*H of Ph), 129.0–128.6 (m, *C*Hs of Phs), 48.3 (s, *C*HMe$_2$), 44.6 (d, $J_{C\text{-}P}$ = 26.4 Hz, CH_2CH_2P), 33.6 (d, $J_{C\text{-}P}$ = 14.3 Hz, CH_2CH_2P), 26.3 (s, CH*Me*$_2$) ppm. $^{31}P\{^1H\}$ NMR (C_6D_6, 162.0 MHz, 298 K; Figure S6): δ –21.8 (s, sat, $J_{P\text{-}Sn}$ = 34 Hz) ppm. $^{119}Sn\{^1H\}$ NMR (C_6D_6, 149.2 MHz, 298 K; Figure S6): δ –154 (br s) ppm.

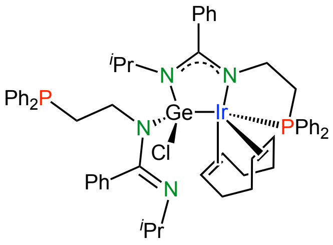

[Ir{κ^3*Ge,N,P*-GeCl(bzamP)$_2$(cod)] (3a): Germylene **2a** (49 mg, 0.06 mmol) was dissolved in C_6D_6 (0.5 mL) and the resulting solution was added to a vial containing $[IrCl(cod)]_2$ (20 mg, 0.03 mmol). The resulting orange suspension was stirred for 1 h to give a yellow solution. ^{1}H and $^{31}P\{^1H\}$ NMR analyses showed the almost quantitative formation of **3a**. The solvent was vacuum-dried

and the solid residue was washed with hexane (1 mL) to give **3a** as a yellow solid (56 mg, 81% yield). (+)-ESI-Q-TOF: *m/z* 1157.3538; calcd. for $C_{56}H_{65}ClGeIrN_4P_2$: 1157.3216 [M + H]$^+$. ^{1}H NMR (C_6D_6, 400.5 MHz, 298 K; Figure 7): δ 7.65 (m, 3 H, C*H*s of Phs), 7.44 (br s, 2 H, C*H*s of Phs), 7.32–6.79 (m, 25 H, C*H*s of Phs), 5.15 (t, J = 6.0 Hz, 1 H, C*H* of cod), 4.14 (vbr s, 1 H, C*H* of cod), 3.60–2.75 (m, 11 H), 2.33 (m, 1 H), 2.24–1.98 (m, 2 H), 1.78–1.58 (m, 9 H), 1.55 (d, J = 6.0 Hz, 3 H, Me of CH*Me*$_2$), 1.48 (d, J = 6.0 Hz, 3 H, Me of CH*Me*$_2$), 1.30 (m, 2 H), 0.97 (m, 1 H) ppm.^{13}C{^{1}H} NMR (C_6D_6, 100.7, 298 K; Figure S7): δ 171.4 (s, N*C*N), 162.5 (s, N*C*N), 144.8 (d, $J_{C\text{-}P}$ = 37.7 Hz, *C* of Ph), 139.9 (d, $J_{C\text{-}P}$ = 12.2 Hz, *C* of Ph), 137.8 (d, $J_{C\text{-}P}$ = 12.6 Hz, *C* of Ph), 137.1 (d, $J_{C\text{-}P}$ = 35.4 Hz, *C* of Ph), 136.2 (s, *C*H of Ph), 136.0 (d, $J_{C\text{-}P}$ = 14.3 Hz, *C*H of Ph), 135.5 (s, *C* of Ph), 133.2 (d, $J_{C\text{-}P}$ = 19.4 Hz, *C*H of Ph), 132.7 (d, $J_{C\text{-}P}$ = 18.7 Hz, *C*H of Ph), 130.6 (d, $J_{C\text{-}P}$ = 9.2 Hz, *C*H of Ph), 130.0 (s, *C*H of Ph), 129.6–127.3 (m, *C*Hs of Phs), 66.8 (s, *C*H of cod), 64.6 (s, *C*HMe$_2$), 60.8 (d, $J_{C\text{-}P}$ = 8.0 Hz), 58.7 (d, $J_{C\text{-}P}$ = 27.2 Hz), 55.4 (s), 52.1 (br s), 50.4 (s), 43.4 (d, $J_{C\text{-}P}$ = 32.2 Hz), 38.2 (d, $J_{C\text{-}P}$ = 29.2 Hz), 34.1 (s), 33.3–32.6 (m), 31.9 (s), 26.1 (s, Me of CH*Me*$_2$), 25.6 (s, Me of CH*Me*$_2$), 25.3 (br s, Me of CH*Me*$_2$), 21.5 (br s, Me of CH*Me*$_2$) ppm. ^{31}P{^{1}H} NMR (C_6D_6, 162.1 MHz, 298 K; Figure S8): δ 21.0 (s), –20.1 (s) ppm.

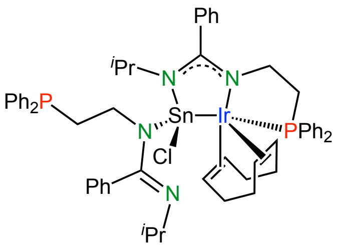

[Ir{κ^3*Sn*,*N*,*P*-SnCl(bzamP)$_2$}(cod)] (3b): [IrCl(cod)]$_2$ (48 mg, 0.07 mmol) was added to a solution of stannylene **2b** (124 mg, 0.14 mmol) in C_6D_6 (0.5 mL). The resulting orange solution was stirred for 1 h. ^{1}H and ^{31}P{^{1}H} NMR analyses showed the almost quantitative formation of **3b**. The solvent was vacuum-dried and the solid residue was washed with pentane (3x2 mL) to give **3b** as a yellow solid (164 mg, 98%). (+)-ESI-Q-TOF: *m/z* 1121.3810; calcd. for $C_{50}H_{60}IrN_4O_2P_2Sn$: 1121.2845 [*M* – Cl – cod + 2 MeOH]$^+$. ^{1}H NMR (C_6D_6, 400.5 MHz, 298 K; Figure S9): δ 7.76 (d, J = 6.0 Hz, 1 H, C*H* of Ph), 7.52–6.65 (m, 29 H, C*H*s of Phs), 4.48 (t, J = 7.5 Hz, 1 H, C*H* of cod), 3.91 (m, 1 H), 3.76–3.45 (m, 4 H), 3.40–2.87 (m, 7 H), 2.47 (m, 1 H), 2.24 (m, 2 H), 2.13–1.91 (m, 2 H), 1.85 (d, J = 6.0 Hz, 3 H, Me of CH*Me*$_2$), 1.70 (m, 1 H), 1.61–1.42 (m, 9 H, 3 Me of 2 CH*Me*$_2$), 1.32 (m, 2 H), 1.05 (m, 1 H) ppm. ^{13}C{^{1}H} NMR (C_6D_6, 100.7 MHz, 298 K; Figure S9): δ 170.1 (s, N*C*N), 167.6 (s, N*C*N), 143.7 (d, $J_{C\text{-}P}$ = 38.0 Hz, *C* of Ph), 139.9 (d, $J_{C\text{-}P}$ =12.7 Hz, *C* of Ph), 138.9 (d, $J_{C\text{-}P}$ = 37.7 Hz, *C* of Ph), 137.4 (d, $J_{C\text{-}P}$ = 11.8 Hz, *C* of Ph), 134.8 (d, $J_{C\text{-}P}$ = 13.1 Hz, *C*H of Ph), 134.1 (s, *C*H of Ph), 133.5 (d, $J_{C\text{-}P}$ = 20.0 Hz, *C*H of Ph), 132.4 (d, $J_{C\text{-}P}$ =

18.4 Hz, *C*H of Ph), 130.7 (d, $J_{C\text{-}P}$ = 9.5 Hz, *C*H of Ph), 129.6–127.3 (m, *C*Hs of Phs), 64.8 (s, *C*H of cod), 63.4 (s, *C*HMe$_2$), 61.4 (d, $J_{C\text{-}P}$ = 8.0 Hz), 56.4 (s), 56.4 (s), 54.5 (d, $J_{C\text{-}P}$ = 28.2 Hz), 52.4 (s), 48.2 (s), 43.1 (d, $J_{C\text{-}P}$ = 29.2 Hz), 37.4 (d, $J_{C\text{-}P}$ = 30.2 Hz), 36.6 (d, $J_{C\text{-}P}$ = 8.0 Hz), 35.7 (s), 33.4 (d, $J_{C\text{-}P}$ = 16.1 Hz), 31.1 (s), 29.9 (s), 26.3 (s, Me of CH*Me*$_2$), 25.9 (s, Me of CH*Me*$_2$), 25.7 (s, Me of CH*Me*$_2$), 21.6 (s, Me of CH*Me*$_2$) ppm. ^{31}P{^{1}H} NMR (C_6D_6, 121.5 MHz, 298 K; Figure S10): δ 25.2 (s, sat, $J_{P\text{-}Sn}$ = 271 Hz), –21.1 (s) ppm. ^{119}Sn{^{1}H} NMR (C_6D_6, 149.2 MHz, 298 K; Figure S10): δ –262 (d, $J_{Sn\text{-}P}$ = 271 Hz) ppm.

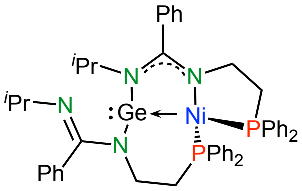

[Ni{κ^4*Ge,N,P,P'*-Ge(bzam)$_2$}] (4a): [Ni(cod)$_2$] (14 mg, 0.05 mmol) and germylene **2a** (41 mg, 0.05 mmol) were dissolved in C_6D_6 (0.5 mL) and the resulting suspension was stirred for 30 min to give a bright red solution. ^{1}H and ^{31}P{^{1}H} NMR analyses only showed the signals of **4a** and free cod. All volatiles were removed *in vacuo* and the solid residue was washed with hexane (2×4 mL) to give **4a** as a red solid (38 mg, 86%). Anal. (%) calcd. for $C_{48}H_{52}GeN_4NiP_2$ (*M* = 878.23): C, 65.65; H, 5.97; N, 6.38; found: C, 65.06; H, 5.97; N, 6.30. ^{1}H NMR (C_6D_6, 400.5 MHz, 298 K; Figure S11): δ 8.36 (t, *J* = 8.0 Hz, 2 H, C*H*s of Phs), 7.78 (t, *J* = 7.0 Hz, 2 H, C*H*s of Phs), 7.44 (d, *J* = 8.0 Hz, 1 H, C*H* of Ph), 7.33–6.47 (m, 24 H, C*H*s of Phs), 5.48–5.13 (m, 2 H), 3.81–3.57 (m, 3 H), 3.34–3.10 (m, 2 H), 2.62 (m, 1 H), 2.11 (m, 1 H), 1.83 (m, 1 H), 1.73 (m, 1 H), 1.44 (d, *J* = 6.7 Hz, 3 H, Me of CH*Me*$_2$), 1.33 (d, *J* = 6.0 Hz, 3 H, Me of CH*Me*$_2$), 1.29 (d, *J* = 5.8 Hz, 3 H, Me of CH*Me*$_2$), 1.25 (d, *J* = 6.1 Hz, 3 H, Me of CH*Me*$_2$) ppm. ^{13}C{^{1}H} NMR (C_6D_6, 100.7, 298 K; Figure S11): δ 165.3 (d, $J_{C\text{-}P}$ = 6.8 Hz, N*C*N), 160.6 (s, N*C*N), 140.7 (dd, $J_{C\text{-}P}$ = 32.3 and 5.9 Hz, *C* of Ph), 139.7 (br s, *C* of Ph), 139.3 (s, *C* of Ph), 138.1 (d, $J_{C\text{-}P}$ = 38.5 Hz, *C* of Ph), 136.8 (d, $J_{C\text{-}P}$ = 20.3 Hz, *C* of Ph), 135.8 (d, $J_{C\text{-}P}$ = 12.8 Hz, *C*H of Ph), 134.8 (d, $J_{C\text{-}P}$ = 21.1 Hz, *C* of Ph), 134.4 (d, $J_{C\text{-}P}$ = 15.4 Hz, *C*H of Ph), 132.0 (d, $J_{C\text{-}P}$ = 12.7 Hz, *C*H of Ph), 130.9 (d, $J_{C\text{-}P}$ = 10.8 Hz, *C*H of Ph), 129.7 (s, *C*H of Ph), 129.4 (s, *C*H of Ph), 129.0 (s, *C*H of Ph), 128.9–127.2 (m, *C*Hs of Phs), 127.0 (s, *C*H of Ph), 125.9 (s, *C*H of Ph), 51.3 (s, *C*HMe$_2$), 50.4 (d, $J_{C\text{-}P}$ = 10.7 Hz, CH$_2$*C*H$_2$P), 50.2 (s, *C*HMe$_2$), 49.7 (br s, *C*H$_2$CH$_2$P), 33.0 (d, $J_{C\text{-}P}$ = 19.0 Hz, CH$_2$*C*H$_2$P), 26.9 (s, Me of CH*Me*$_2$), 26.8 (s, Me of CH*Me*$_2$), 26.5 (s, Me of CH*Me*$_2$), 26.2 (d, $J_{C\text{-}P}$ = 19.0 Hz, *C*H$_2$CH$_2$P), 23.9 (s, Me of CH*Me*$_2$) ppm. ^{31}P{^{1}H} NMR (C_6D_6, 121.5 MHz, 298 K; Figure S12): δ 43.7 (d, $J_{P\text{-}P}$ = 27 Hz), 18.6 (d, $J_{P\text{-}P}$ = 27 Hz) ppm.

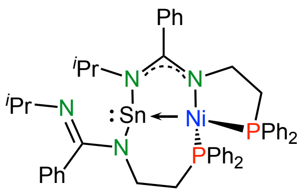

[Ni(κ^4*Sn,N,P,P*-Sn(bzam)$_2$] (4b): [Ni(cod)$_2$] (16 mg, 0.06 mmol) and stannylene **2b** (52 mg, 0.06 mmol) were dissolved in C_6D_6 (0.5 mL) and the resulting suspension was stirred for 30 min to give a dark red solution. ^{1}H and ^{31}P{^{1}H} NMR analyses (Figure S13) showed the almost quantitative formation of **4b**. However, this product always decomposed during the workup and it could not be isolated as a pure solid. The following NMR data correspond to the crude reaction outcome of a reaction carried out in C_6D_6 in an NMR tube. ^{1}H NMR (C_6D_6, 300.1 MHz, 298 K; Figure S13): δ 8.25 (t, J = 9.0 Hz, 2 H, C*H*s of Phs), 7.64 (t, J = 9.0 Hz, 2 H, C*H*s of Phs), 7.48 (d, J = 9.0 Hz, 1 H, C*H* of Ph), 7.34–6.45 (m, 24 H, C*H*s of Ph), 5.52–5.30 (m, 2 H), 4.16 (m, 1 H), 3.97 (m, 1 H), 3.53 (m, 1 H), 3.32 (m, 1 H), 2.95 (m, 1 H), 2.67 (m, 1 H), 2.16–1.76 (m, 3 H), 1.47–1.20 (m, 12 H, 2 CH*Me*$_2$) ppm. ^{13}C{^{1}H} NMR (C_6D_6, 100.7 MHz, 298 K; Figure S13): δ 169.1 (s, N*C*N), 162.9 (s, N*C*N), 140.5 (dd, $J_{C\text{-}P}$ = 32.6 and 10.8 Hz, *C* of Ph), 140.2 (s, *C* of Ph), 139.3 (s, *C* of Ph), 138.3 (d, $J_{C\text{-}P}$ = 40.4 Hz, *C* of Ph), 136.8 (d, $J_{C\text{-}P}$ = 20.5 Hz, *C* of Ph), 135.4 (d, $J_{C\text{-}P}$ = 12.0 Hz, *C*H of Ph), 134.7 (d, $J_{C\text{-}P}$ = 16.8 Hz, *C* of Ph), 133.7 (d, $J_{C\text{-}P}$ = 13.9 Hz, *C*H of Ph), 132.6 (d, $J_{C\text{-}P}$ = 13.3 Hz, *C*H of Ph), 130.8 (d, $J_{C\text{-}P}$ = 10.0 Hz, *C*H of Ph), 129.7 (s, *C*H of Ph), 129.2 (d, $J_{C\text{-}P}$ = 9.3 Hz, *C*H of Ph), 128.7–127.5 (m, *C*Hs of Phs), 127.3 (s, *C*H of Ph), 127.2 (s, *C*H of Ph), 127.0 (s, *C*H of Ph), 126.0 (s, *C*H of Ph), 51.2 (s, *C*HMe$_2$), 50.8 (br s, CH$_2$*C*H$_2$P), 50.6 (s, *C*HMe$_2$), 50.0 (s, *C*H$_2$CH$_2$P), 33.0 (d, $J_{C\text{-}P}$ = 19.0 Hz, CH$_2$*C*H$_2$P), 27.3 (s, Me of CH*Me*$_2$), 26.9 (s, Me of CH*Me*$_2$), 26.8 (s, Me of CH*Me*$_2$), 28.8–27.5 (m, *C*H$_2$CH$_2$P overlapped with *C*H$_2$s of free cod), 24.4 (s, Me of CH*Me*$_2$) ppm. ^{31}P{^{1}H} NMR (C_6D_6, 121.5 MHz, 298 K; Figure S14): δ 37.1 (d, sat, $J_{P\text{-}P}$ = 32 Hz, $J_{P\text{-}Sn}$ = 88 Hz), 18.3 (d, sat, $J_{P\text{-}P}$ = 32 Hz, $J_{P\text{-}Sn}$ = 40 Hz) ppm. ^{119}Sn{^{1}H} NMR (C_6D_6, 149.2 MHz, 298 K; Figure S14): δ 455 (dd, $J_{P\text{-}Sn}$ = 76 and 44 Hz) ppm

X-Ray Diffraction Analyses. Crystals of **2a**, **2b**·(C_7H_8), **3a**·(C_6H_{14}), **3b**·0.5($C_4H_{10}O$) and **4a**·(C_7H_8) were analyzed by X-ray diffraction. A selection of crystal, measurement and refinement data is given in Table S1. Diffraction data were collected on an Oxford Diffraction Xcalibur Onyx Nova single crystal diffractometer with CuKα radiation. Empirical absorption corrections were applied using the SCALE3 ABSPACK algorithm as implemented in CrysAlisPro RED.[S3] The structures were solved with SIR-97.[S4] Isotropic and full matrix anisotropic least square refinements were carried out using SHELXL.[S5] One *iso*propyl group (C75) of **2a** was disordered over two positions with a 75:25 occupancy ratio, requiring restraints on its geometrical and thermal parameters.

The toluene solvent molecule found in the asymmetric unit of **2b**·(C_7H_8) was disordered over two positions with a 79:21 occupancy ratio, requiring restraints on its geometrical and thermal parameters. The solvent molecules found in the asymmetric unit of **3a**·(C_6H_{14}), which were severely disordered, were modelled as two half hexane molecules with an equal occupancy ratio (each one disordered into two positions with an equal occupancy ratio) and required restraints on their geometrical and thermal parameters. The half ether solvent molecule found in the asymmetric unit of **3b**·0.5($C_4H_{10}O$) was disordered about a center of symmetry and required restraints on its geometrical and thermal parameters. The WINGX program system[S6] was used throughout the structure determinations. The molecular plots were made with MERCURY.[S7]

Theoretical Calculations. The structure optimization of **4a** was performed with the *Gaussian09* suite of programs,[S8] using the wB97XD functional,[S9] which includes the second generation of Grimme's dispersion interaction correction.[S10] The Stuttgart-Dresden relativistic effective core potentials and the associated basis sets (SΔΔ) was used for the Ni, Ge and Sn atoms.[S11] The basis set used for the remaining atoms was the cc-pVDZ.[S12] Frequency calculations confirmed the optimized structure as energy minimum (zero imaginary eigenvalues). Gibbs energies were computed at 298.15 K and 1.0 atm. Orbital calculations were performed with the NBO package.[S13]

References

S1 Kumar, P. R.; Upreti, S.; Singh, A. K. *Polyhedron* **2008**, *27*, 2610–1622.

S2 Takahashi, H.; Fukami, T.; Kojima, H.; Yamakawa, T.; Takahashi, H.; Sakamoto, T.; Nishimura, T.; Nakamura, M.; Yosizumi, T.; Miiyama, K.; Ohtake, N.; Hayama, T. *Tetrahedron* **2005**, *61*, 3473–3481.

S3 *CrysAlisPro RED*, version 1.171.38.46: Oxford Diffraction Ltd., Oxford, UK, 2015.

S4 *SIR-97*: Altomare, A.; Burla, M. C.; Camalli, M.; Cascarano, G. L.; Giacovazzo, C.; Guagliardi, A.; Moliterni, A. G. C.; Polidori, G.; Spagna, R. *J. Appl. Crystallogr.* **1999**, *32*, 115–119.

S5 *SHELXL-2014*: Sheldrick, G. M. *Acta Cryst.* **2008**, *A64*, 112–122.

S6 *WINGX*, version 2021.3: Farrugia, L. *J. Appl. Crystallogr.* **2012**, *45*, 849–854.

S7 *MERCURY*, version 2022.2.0 (build 353591): Cambridge Crystallographic Data Centre, Cambridge, UK, 2022.

S8 Frisch, M. J.; Trucks, G. W.; Schlegel, H. B.; Scuseria, G. E.; Robb, M. A.; Cheeseman, J. R.; Scalmani, G.; Barone, V.; Mennucci, B.; Petersson, G. A.; Nakatsuji, H.; Caricato, M.; Li, X.; Hratchian, H. P.; Izmaylov, A. F.; Bloino, J.; Zheng, G.; Sonnenberg, J. L.; Hada, M.; Ehara, M.; Toyota, K.; Fukuda, R.; Hasegawa, J.; Ishida, M.; Nakajima, T.; Honda, Y.; Kitao, O.; Nakai, H.; Vreven, T.; Montgomery, J. A., Jr.; Peralta, J. E.; Ogliaro, F.; Bearpark, M.; Heyd, J. J.; Brothers, E.; Kudin, K. N.; Staroverov, V. N.; Kobayashi, R.; Normand, J.; Raghavachari, K.; Rendell, A.; Burant, J. C.; Iyengar, S. S.; Tomasi, J.; Cossi, M.; Rega, N.; Millam, J. M.; Klene, M.; Knox, J. E.; Cross, J. B.; Bakken, V.; Adamo, C.; Jaramillo, J.; Gomperts, R.; Stratmann, R. E.; Yazyev, O.; Austin, A. J.; Cammi, R.; Pomelli, C.; Ochterski, J. W.; Martin, R. L.; Morokuma, K.; Zakrzewski, V. G.; Voth, G. A.; Salvador, P.; Dannenberg, J. J.; Dapprich, S.; Daniels, A. D.; Farkas, O.; Foresman, J. B.; Ortiz, J. V.; Cioslowski, J.; Fox, D. J. *Gaussian 09*, revision A.01; Gaussian, Inc.: Wallingford, CT, **2009**.

S9 Chai, J.-D.; Head-Gordon, M. *Phys. Chem. Chem. Phys.* **2008**, *10*, 6615–6620.

S10 (a) Ehrlich, S.; Moellmann, J.; Grimme, S. *Acc. Chem. Res.* **2013**, *46*, 916–926. (b) Grimme, S. *Comp. Mol. Sci.* **2011**, *1*, 211–228; (c) Schwabe, T.; Grimme, S. *Acc. Chem. Res.* **2008**, *41*, 569–579.

S11 (a) Dolg, M.; Wedig U.; Stoll, H.; Preuss, H. *J. Chem. Phys.* **1987**, 86, 866. (b) Martin, J. M. L.; Sundermann, A. *J. Chem. Phys.* **2001**, 114, 3408–3420.

S12 Dunning, T. H. *J. Chem. Phys*. **1989**, *90*, 1007–1023.

S13 (a) Foster, J. P.; Weinhold, F. *J. Am. Chem. Soc*. **1980**, *102*, 7211–7218. (b) Reed, A. E.; Weinhold, F. *J. Chem. Phys*. **1985**, *83*, 1736–1740. (c) Reed, A. E.; Weinstock R. B.; Weinhold, F. *J. Chem. Phys*. **1985**, *83*, 735–746. (d) Reed, A. E.; Curtiss, L. A.; Weinhold, F. *Chem. Rev.* **1988**, *88*, 899–926.

Table S1. Crystal, measurement and refinement data for the compounds studied by X-ray diffraction.

	**2a**	**2b**·(C_7H_8)	**3a**·(C_6H_{14})	**3b**·0.5($C_4H_{10}O$)	**4a**·(C_7H_8)
formula	$(C_{48}H_{52}GeN_4P_2)_2$	$C_{48}H_{52}N_4P_2Sn$·(C_7H_8)	$C_{56}H_{64}ClGeIrN_4P_2$ (C_6H_{14})	$C_{56}H_{64}ClIrN_4P_2Sn$·0.5($C_4H_{10}O$)	$C_{48}H_{52}GeN_4NiP_2$·(C_7H_8)
fw	1638.93	957.70	1241.46	1238.45	970.31
cryst syst	triclinic	triclinic	triclinic	triclinic	triclinic
space group	*P*–1	*P*–1	*P*–1	*P*–1	*P*–1
a, Å	10.7299(2)	11.5569(3)	12.3295(7)	12.6360(8)	12.0252(5)
b, Å	19.8496(5)	13.5339(4)	13.4706(5)	13.7309(10)	13.2722(6)
c, Å	21.1239(4)	17.0423(5)	18.4751(8)	16.7785(9)	17.0576(8)
α, deg	83.630(2)	107.419(2)	85.753(3)	99.751(5)	85.506(4)
β, deg	89.228(2)	93.002(2)	88.936(4)	96.473(5)	89.494(3)
γ, deg	82.657(2)	93.474(2)	77.325(4)	104.191(6)	64.733(4)
V, $Å^3$	4434.6(2)	2531.7(1)	2985.4(2)	2745.0(3)	2453.5(2)
Z	2	2	2	2	2
F(000)	1720	996	1268	1246	1016
D_{calcd}, g cm^{-3}	1.227	1.256	1.381	1.498	1.313
μ, mm^{-1} (Cu Kα)	1.893	4.901	6.090	9.552	2.151
cryst size, mm	0.28 x 0.21 x 0.11	0.38 x 0.21 x 0.16	0.22 x 0.13 x 0.03	0.29 x 0.20 x 0.17	0.21 x 0.19 x 0.14
T, K	150(2)	150(2)	150(2)	150(2)	150(2)
θ range, deg	2.91 to 69.55	3.43 to 69.50	3.37 to 69.65	2.71 to 69.37	3.70 to 69.65
min./max. *h*, *k*, *l*	–10/12, –23/23, –24/25	–10/13, –16/15, –20/20	–14/14, –16/12, –22/22	–13/14, –15/15, –19/19	–14/11, –16/15, –20/20
no. collected reflns	44516	24949	26157	16986	25288
no. unique reflns	16420	9359	11021	8505	9119
no. reflns with $I > 2\sigma(I)$	14200	8415	9211	6848	7563
no. params/restraints	1023/04	557/39	666/36	636/12	573/0
GOF (on F^2)	1.083	1.038	1.019	1.019	1.029
R_1 (on *F*, $I > 2\sigma(I)$)	0.064	0.042	0.064	0.071	0.041
wR_2 (on F^2, all data)	0.181	0.118	0.180	0.201	0.112
min./max. $\Delta\rho$, e $Å^{-3}$	–0.527/1.291	–1.212/1.041	–1.725/3.929	–1.156/2.693	–0.483/0.752
CCDC dep. no.	2269825	2269826	2269827	2269828	2269829

NMR Spectra

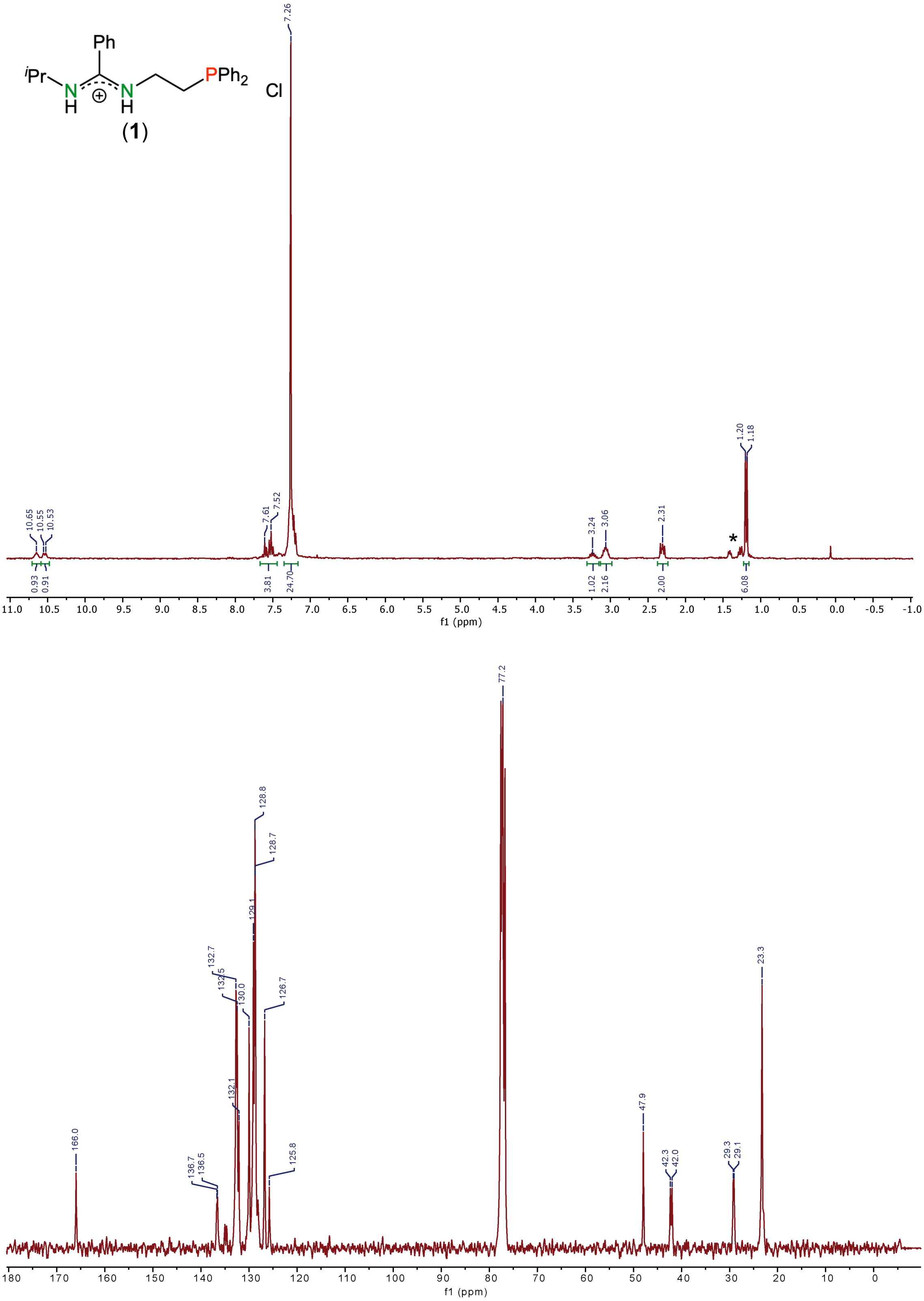

Figure S1. ^{1}H (top, 300.1 MHz) and ^{13}C{^{1}H} (bottom, 75.5 MHz) NMR spectra ($CDCl_3$, 298 K) of [H_2bzamP]Cl (**1**). *Unknown impurity.

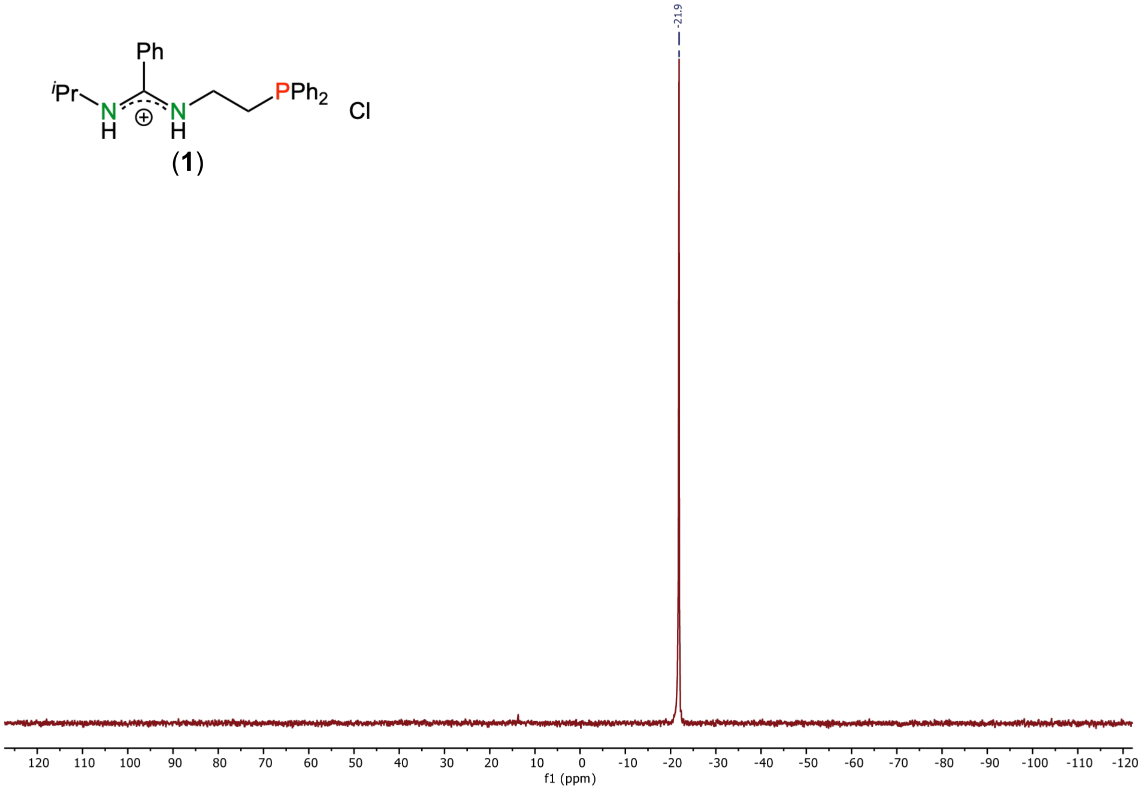

Figure S2. $^{31}P\{^{1}H\}$ NMR spectrum (121.5 MHz, $CDCl_3$, 298 K) of [H_2bzamP]Cl (**1**).

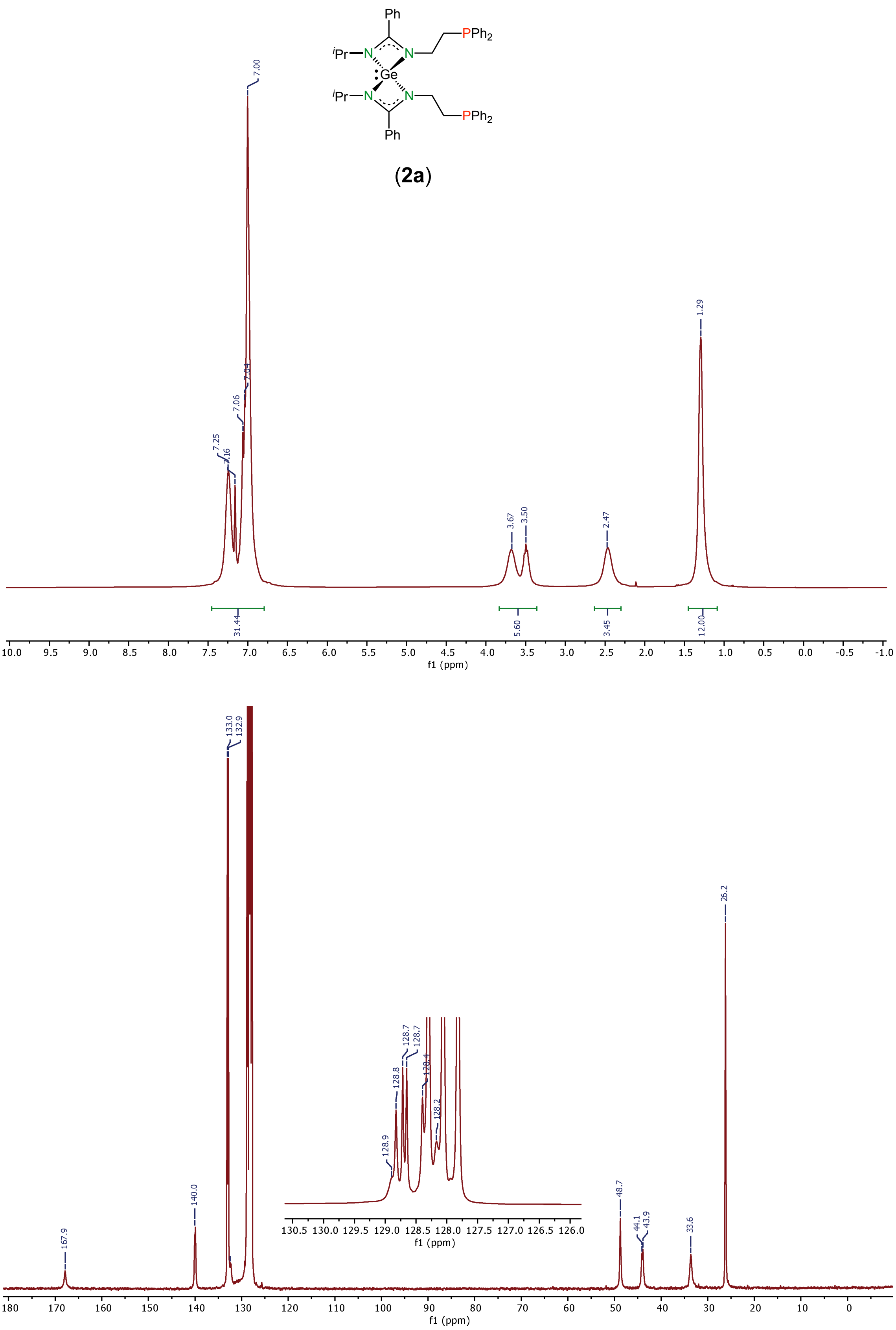

Figure S3. ^{1}H (top, 300.1 MHz) and $^{13}C\{^{1}H\}$ (bottom, 75.5 MHz) NMR spectra (C_6D_6, 298 K) of Ge(bzamP)$_2$ (**2a**).

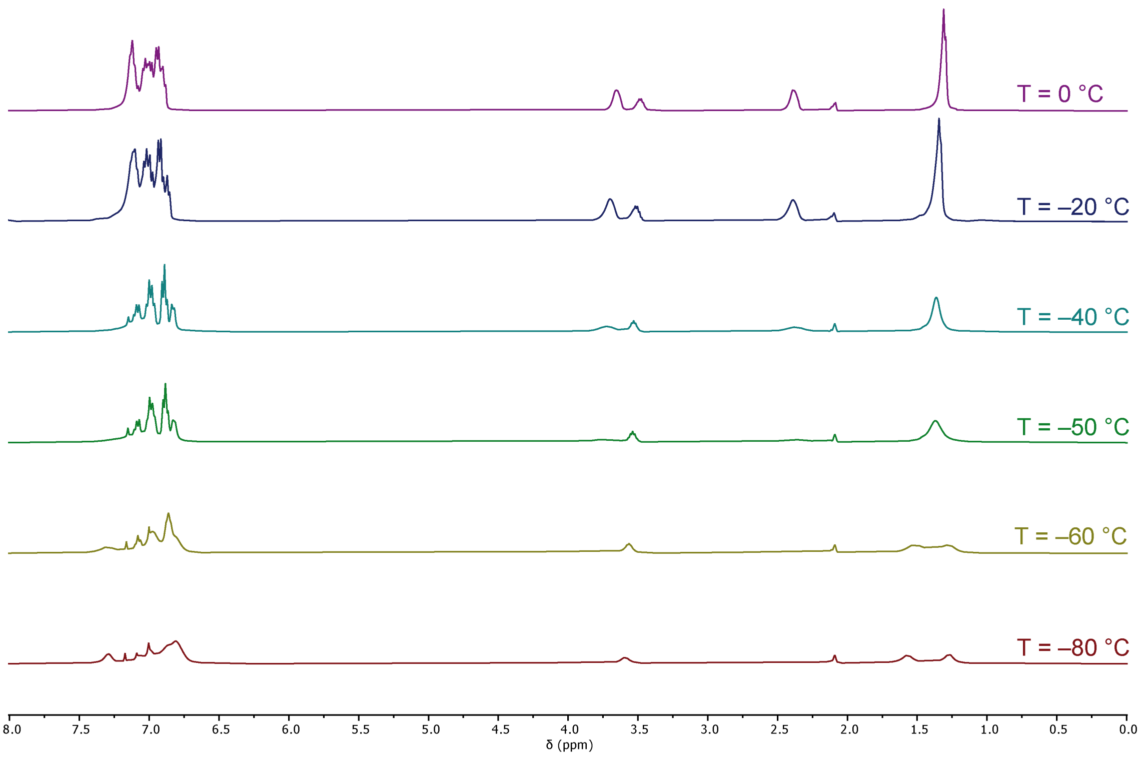

Figure S3a. ^{1}H NMR spectra ($CD_3C_6D_5$, 400.54 MHz) of Ge(bzamP)$_2$ (**2a**) at low temperatures.

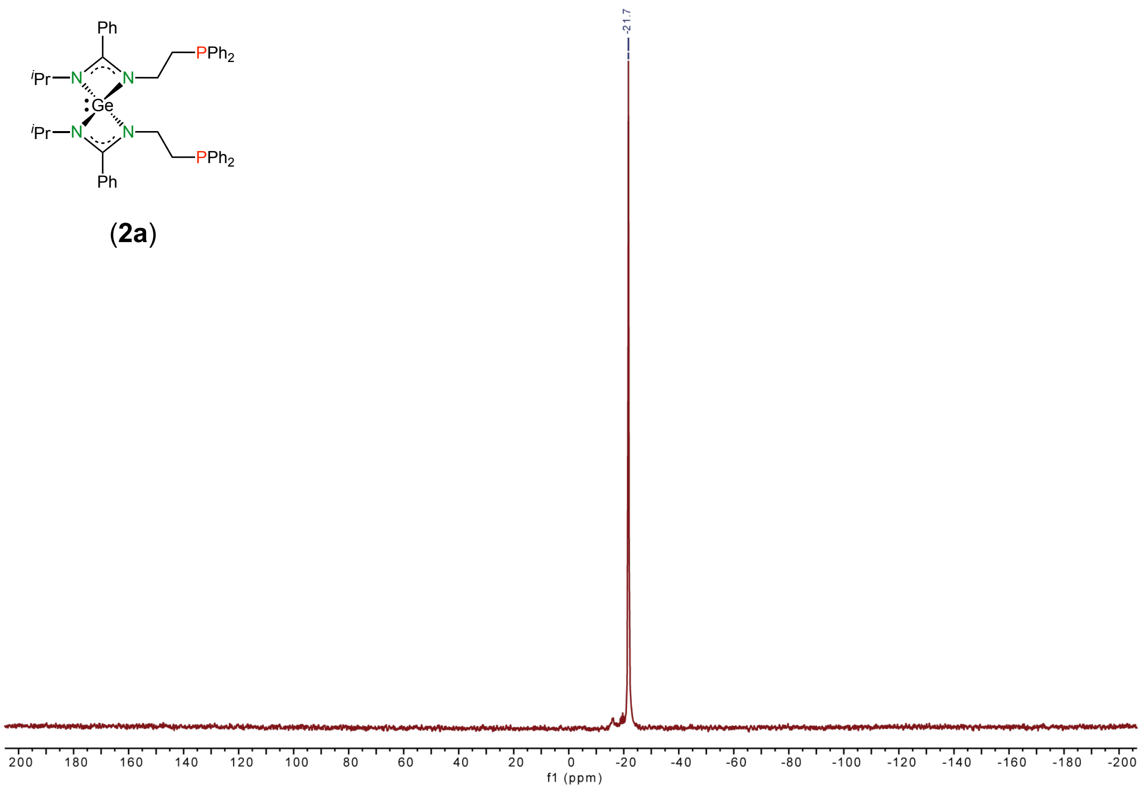

Figure S4. $^{31}P\{^{1}H\}$ NMR spectrum (121.5 MHz, C_6D_6, 298 K) of $Ge(bzamP)_2$ (**2a**).

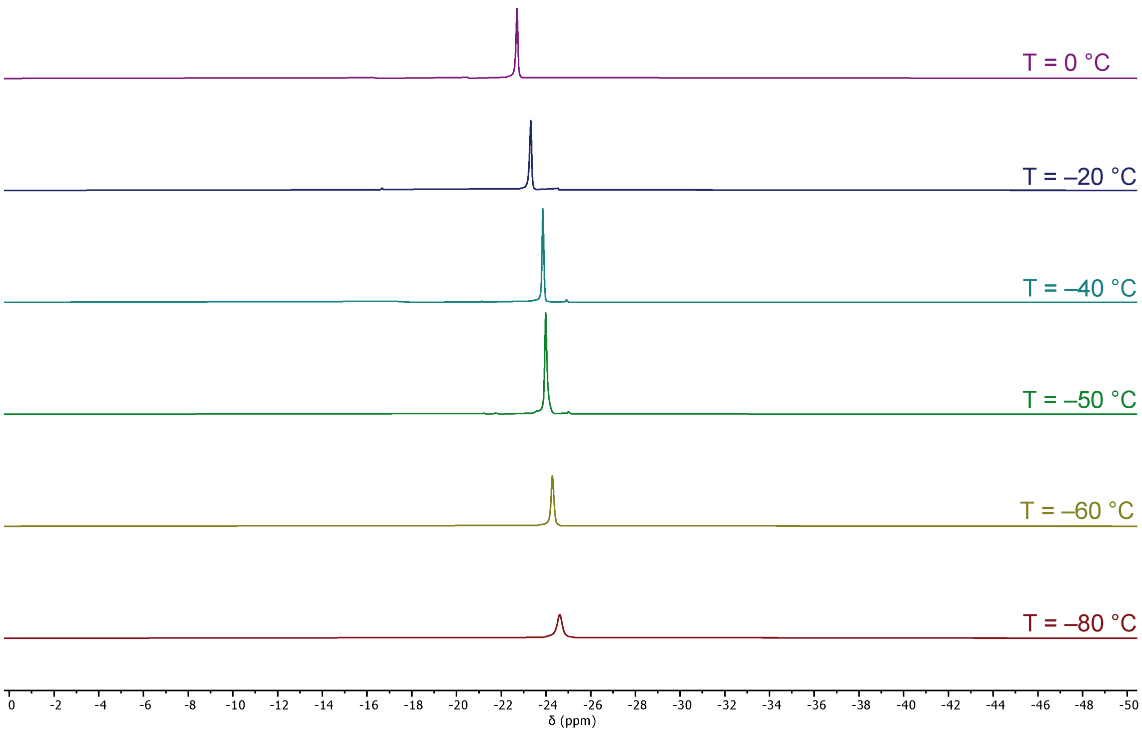

Figure S4a. $^{31}P\{^{1}H\}$ NMR spectra ($CD_3C_6D_5$, 162.14 MHz) of $Ge(bzamP)_2$ (**2a**) at low temperatures.

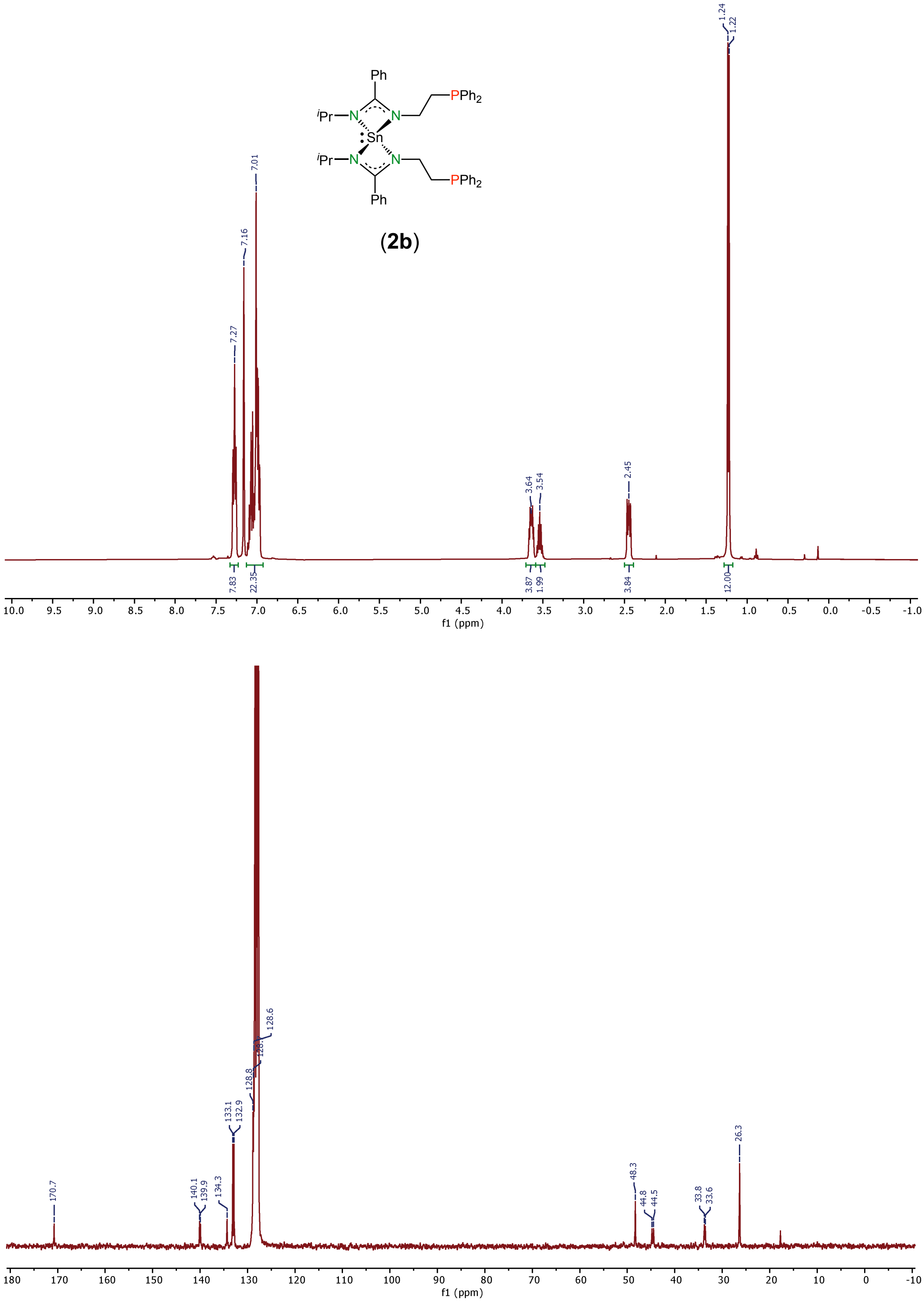

Figure S5. ^{1}H (top, 300.1 MHz) and ^{13}C{^{1}H} (bottom, 75.5 MHz) NMR spectra (C_6D_6, 298 K) of Sn(bzamP)$_2$ (**2b**).

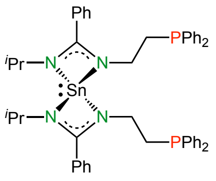

(**2b**)

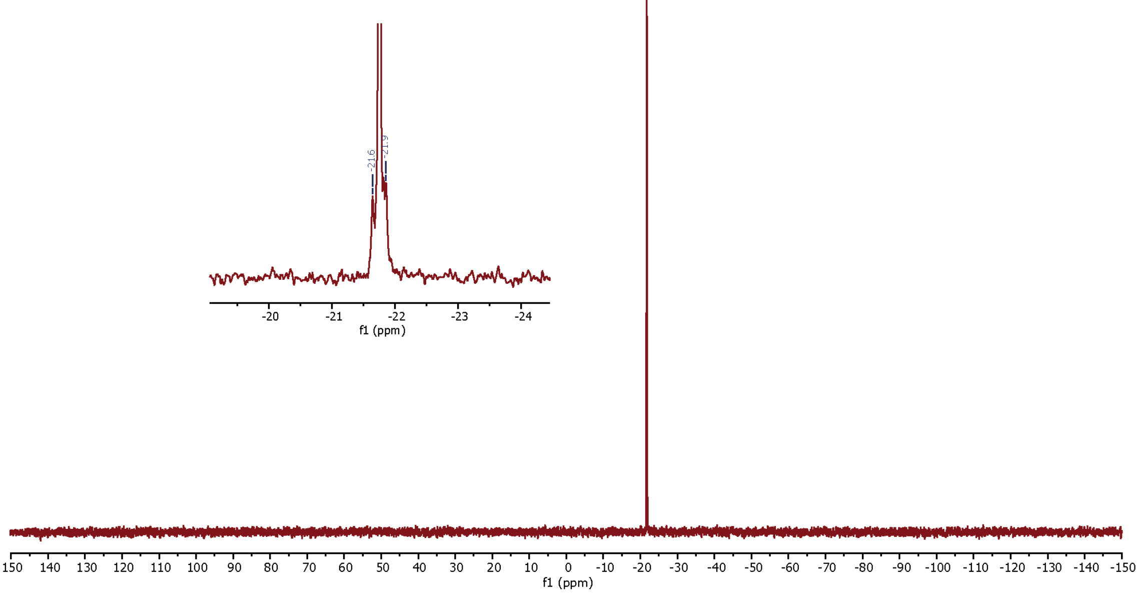

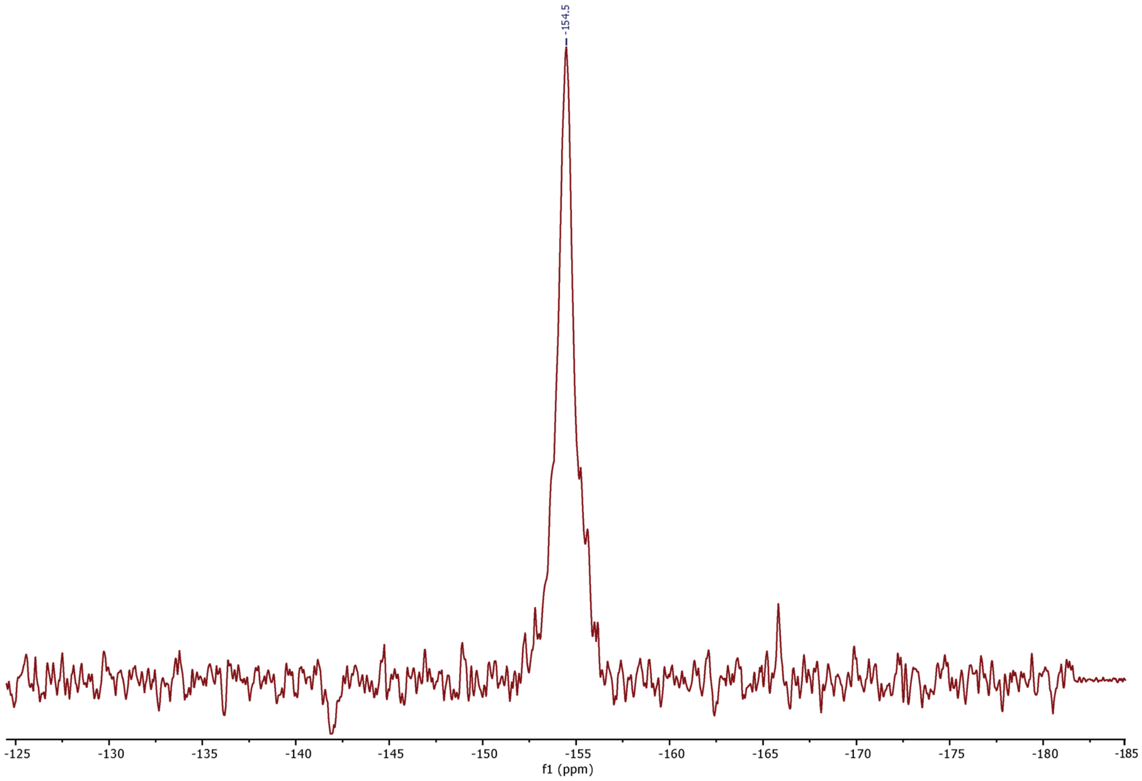

Figure S6. $^{31}P\{^{1}H\}$ (top, 121.5 MHz) and $^{119}Sn\{^{1}H\}$ (bottom, 149.2 MHz) NMR spectra (C_6D_6, 298 K) of $Sn(bzamP)_2$ (**2b**).

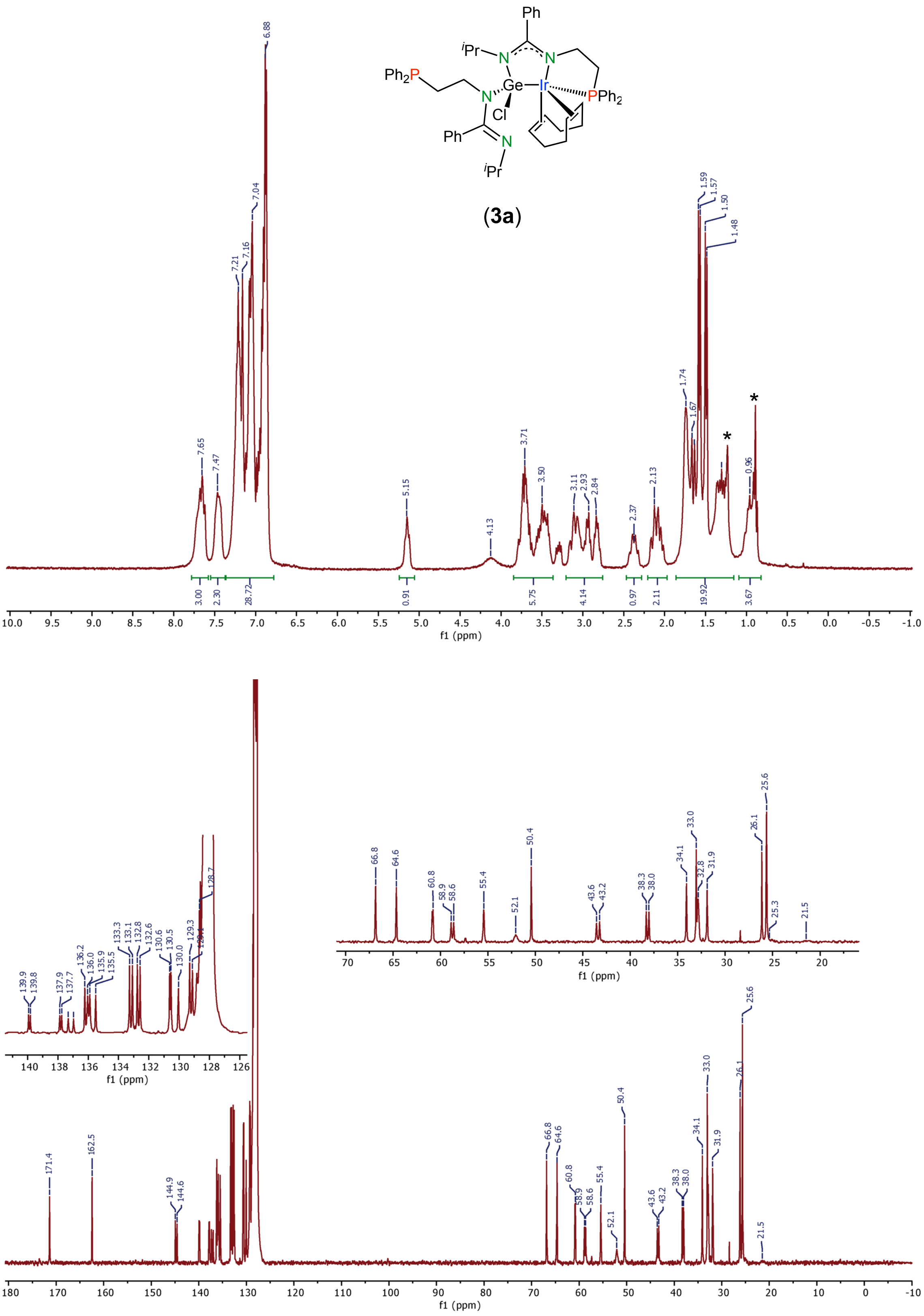

Figure S7. ^{1}H NMR (top, 400.5 MHz, C_6D_6, 298 K, sample washed with *n*-hexane and dried) and ^{13}C{^{1}H} NMR (bottom, 100.7 MHz, C_6D_6, 298 K, dry crude reaction outcome) of [Ir{κ^3*Ge*,*N*,*P*-GeCl(bzamP)$_2$}(cod)] (**3a**). **n*-Hexane.

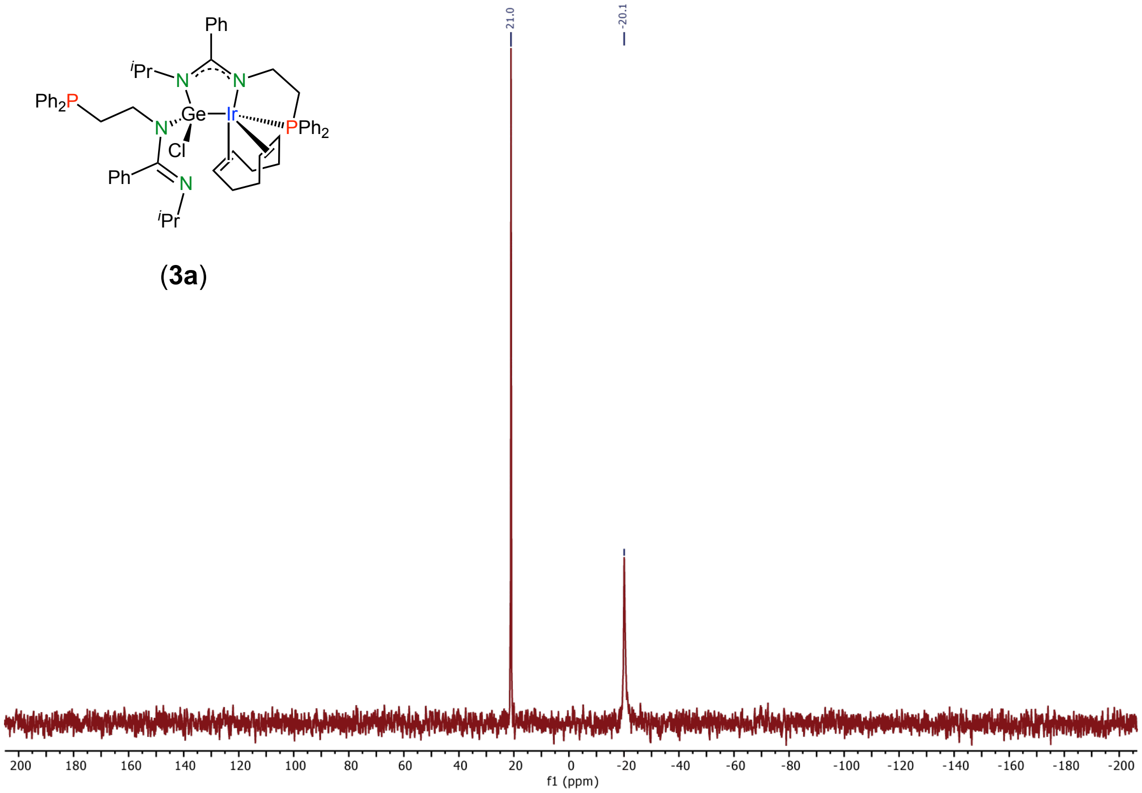

Figure S8. $^{31}P\{^{1}H\}$ NMR spectrum (162.1 MHz, C_6D_6, 298 K) of [Ir{κ^3*Ge*,*N*,*P*-GeCl(bzamP)$_2$}(cod)] (**3a**).

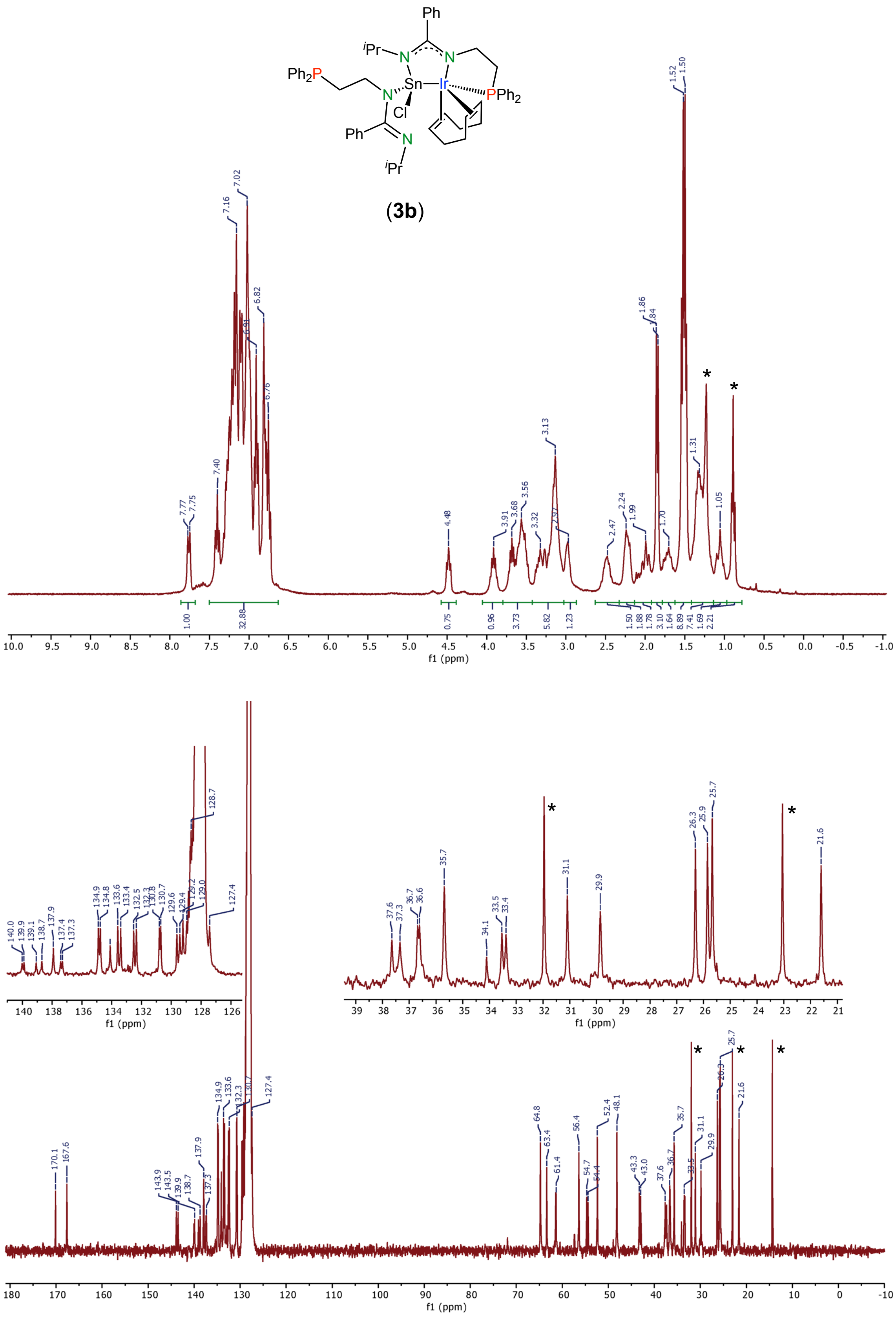

(**3b**)

Figure S9. ^{1}H (top, 400.5 MHz) and ^{13}C{^{1}H} (bottom, 100.7 MHz) NMR spectra (C_6D_6, 298 K) of [Ir{κ^3*Sn*,*N*,*P*-SnCl(bzamP)$_2$}(cod)] (**3b**) (sample washed with *n*-hexane and dried). **n*-Hexane.

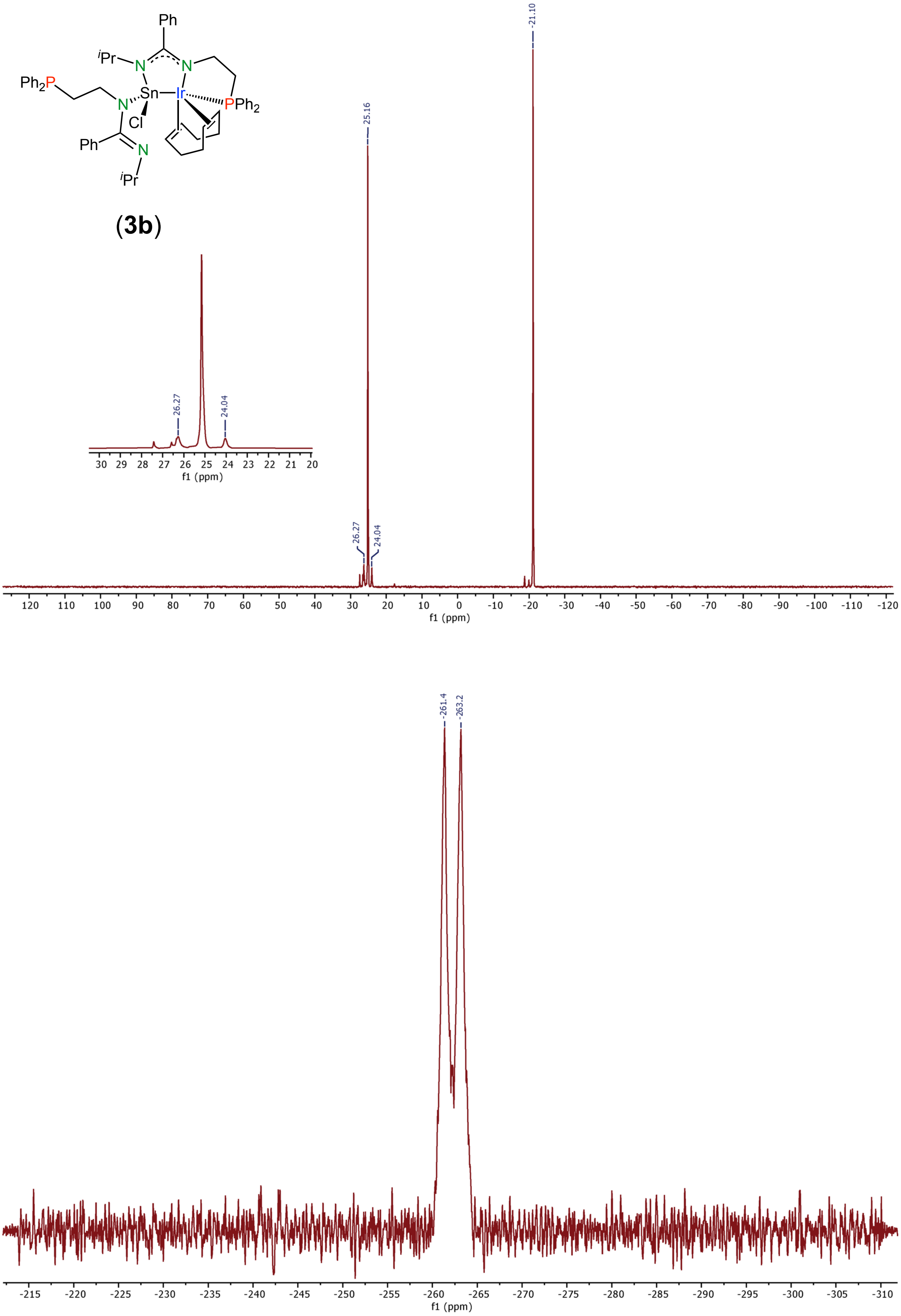

Figure S10. ^{31}P{^{1}H} (top, 121.5 MHz) and ^{119}Sn{^{1}H} (bottom, 149.2 MHz) NMR spectra (C_6D_6, 298 K) of [Ir{κ^3*Sn*,*N*,*P*-SnCl(bzam)$_2$}(cod)] (**3b**).

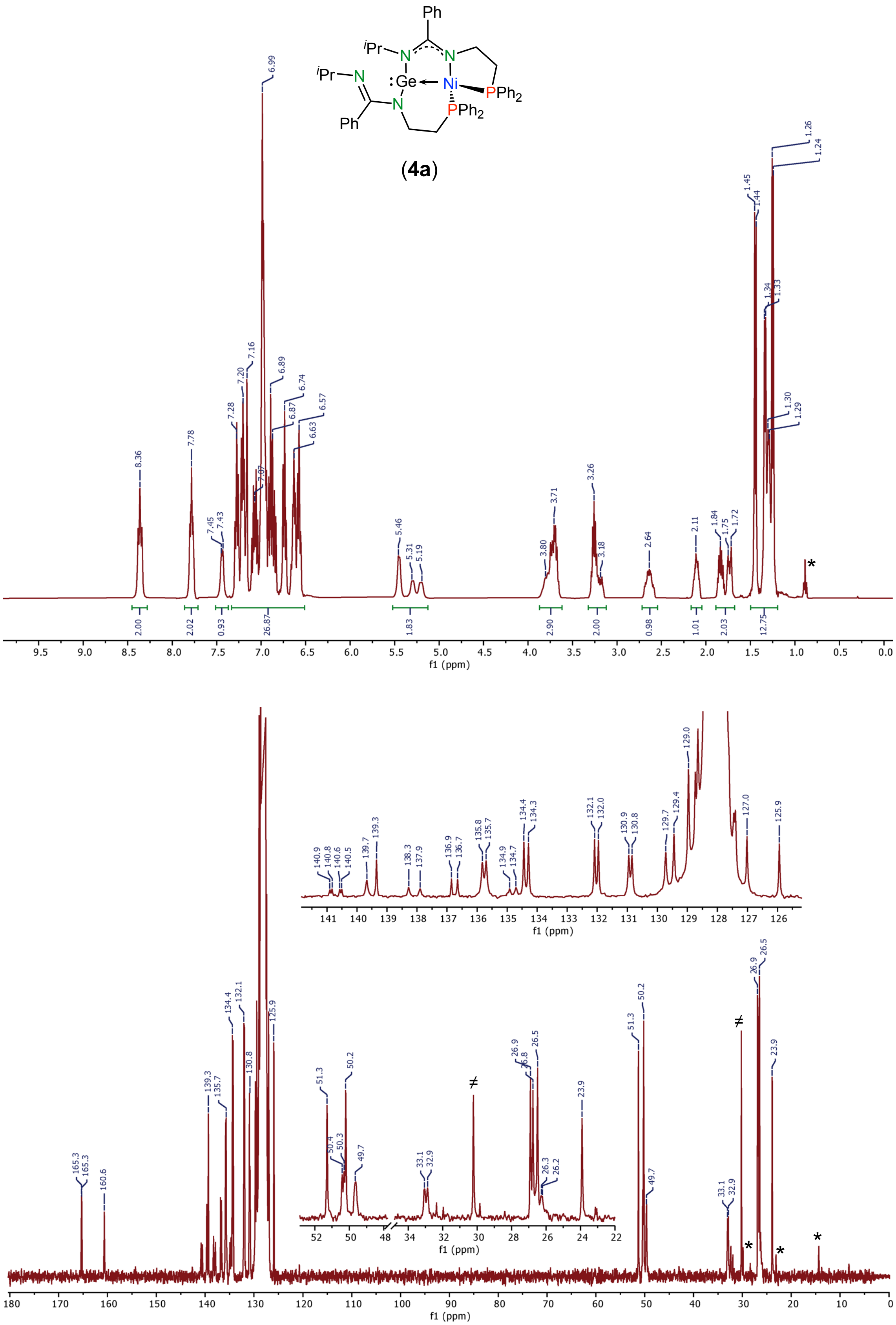

Figure S11. ^{1}H (top 400.5 MHz) and $^{13}C\{^{1}H\}$ (bottom, 100.7 MHz) NMR spectra (C_6D_6, 298 K) of [Ni{κ^{4}*Ge*,*N*,*P*,*P'*-Ge(bzamP)$_2$}] (**4a**). $^{\neq}$Adventitious paraffin oil. *n-Hexane.

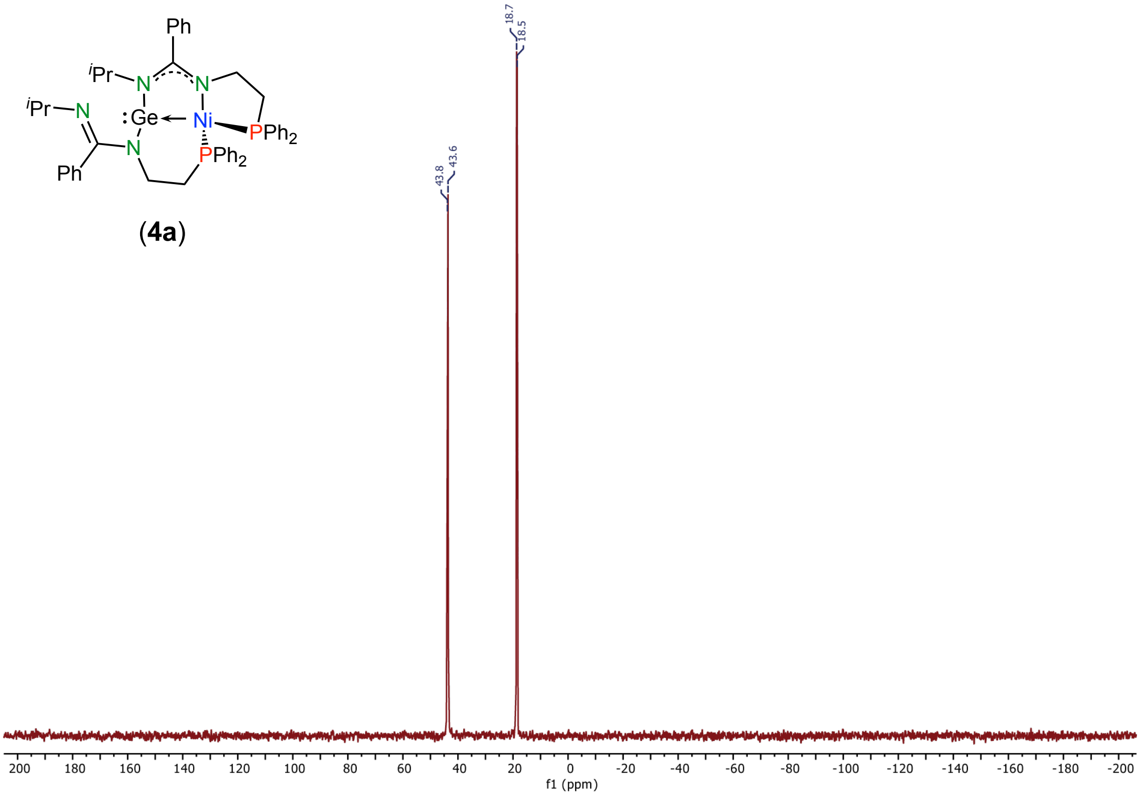

Figure S12. $^{31}P\{^{1}H\}$ NMR spectrum (121.5 MHz, C_6D_6, 298 K) of [Ni{κ^4*Ge*,*N*,*P*,*P'*-Ge(bzamP)$_2$}] (**4a**).

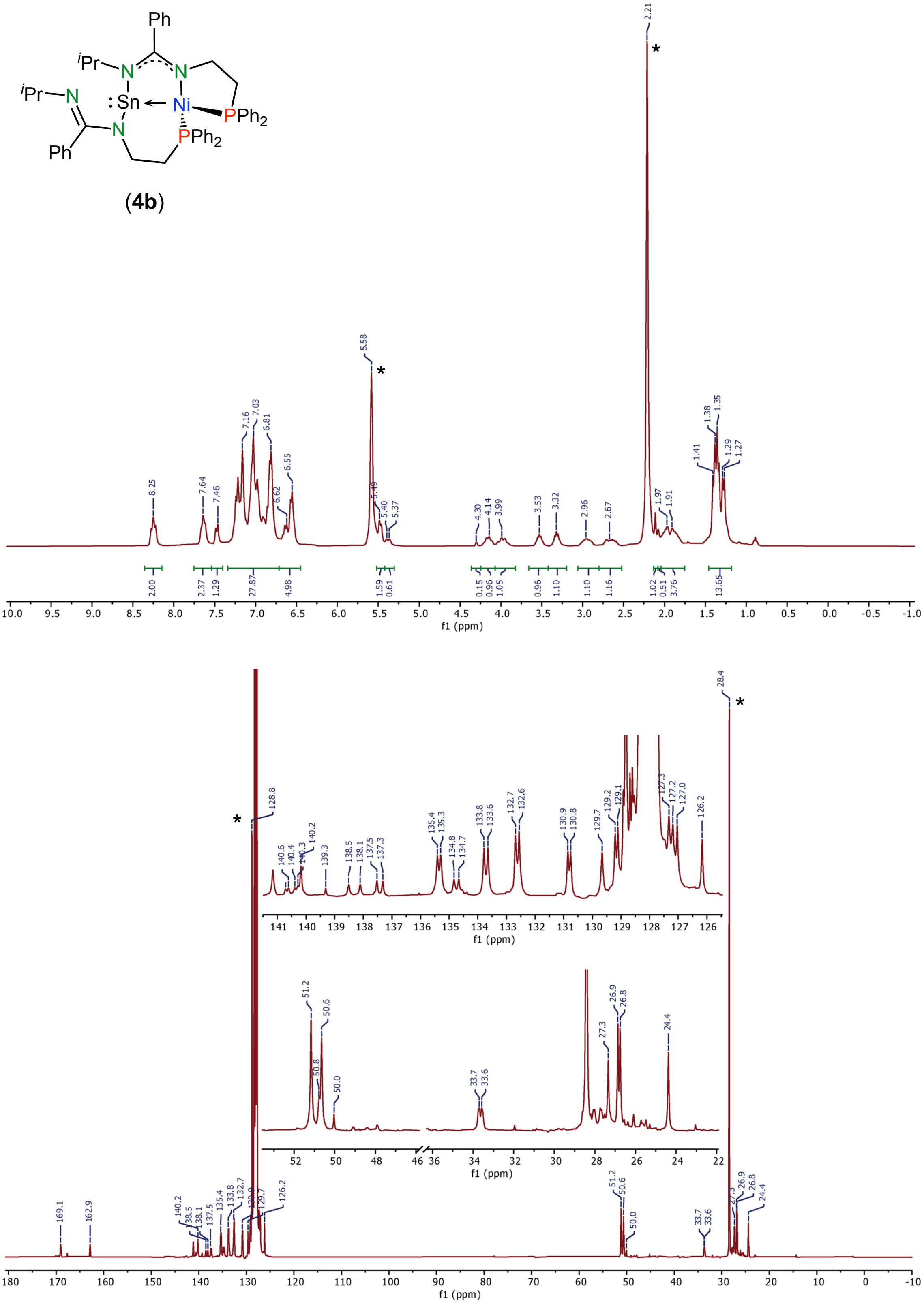

Figure S13. ^{1}H (top 300.1 MHz) and $^{13}C\{^{1}H\}$ (bottom, 100.7 MHz) NMR spectra (C_6D_6, 298 K) of the crude outcome of the reaction of [Ni(cod)$_2$] with **2b** in C_6D_6, showing [Ni{κ^4*Sn*,*N*,*P*,*P'*-Sn(bzamP)$_2$}] (**4b**) and free cod as the major products. *Free cod.

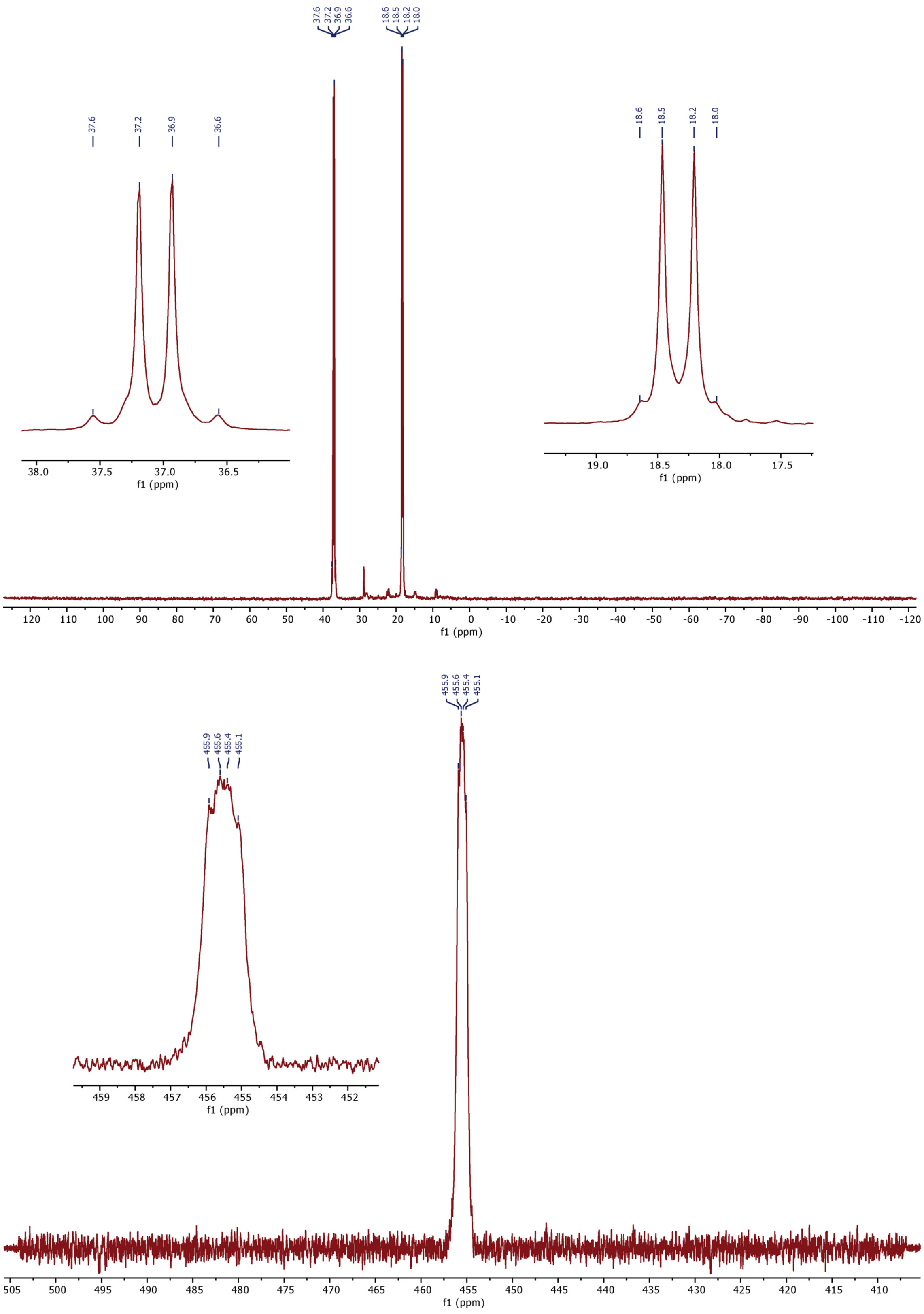

Figure S14. $^{31}P\{^1H\}$ (top, 121.5 MHz) and $^{119}Sn\{^1H\}$ (bottom, 149.2 MHz) NMR spectra (C_6D_6, 298 K) of the crude outcome of the reaction of [Ni(cod)$_2$] with **2b** in C_6D_6, showing [Ni{κ^4*Sn*,*N*,*P*,*P'*-Sn(bzamP)$_2$}] (**4b**) as the major product.
